# Cognitive training, but not EEG-neurofeedback, improves working memory in healthy volunteers

**DOI:** 10.1093/braincomms/fcad101

**Published:** 2023-03-30

**Authors:** Beatrice Barbazzeni, Oliver Speck, Emrah Düzel

**Affiliations:** Faculty of Medicine, Institute of Cognitive Neurology and Dementia Research, Otto-von-Guericke University of Magdeburg, Magdeburg 39120, Germany; ESF-GS ABINEP International Graduate School, Otto-von-Guericke University of Magdeburg, Magdeburg 39106, Germany; Faculty of Natural Science, Department of Biomedical Magnetic Resonance, Otto-von-Guericke University of Magdeburg, Magdeburg 39120, Germany; Leibniz Institute for Neurobiology, Magdeburg 39118, Germany; Center for Behavioral Brain Sciences, Magdeburg 39106, Germany; German Center for Neurodegenerative Diseases, Magdeburg 39120, Germany; Faculty of Medicine, Institute of Cognitive Neurology and Dementia Research, Otto-von-Guericke University of Magdeburg, Magdeburg 39120, Germany; German Center for Neurodegenerative Diseases, Magdeburg 39120, Germany; Neotiv GmbH, Magdeburg 39104, Germany

**Keywords:** cognitive training, working memory, neurofeedback training, monetary reward, alpha suppression

## Abstract

Working memory performance can be influenced by motivational factors, which may be associated with specific brain activities, including suppression of alpha oscillations. We investigated whether providing individuals online feedback about their ongoing oscillations (EEG-neurofeedback) can improve working memory under high and low reward expectancies. We combined working memory training with neurofeedback to enhance alpha suppression in a monetary-rewarded delayed match-to-sample task for visual objects. Along with alpha, we considered the neighbouring theta and beta bands. In a double-blind experiment, individuals were trained over 5 days to suppress alpha power by receiving real-time neurofeedback or control neurofeedback (placebo) in reward and no-reward trials. We investigated (i) whether neurofeedback enhances alpha suppression, (ii) whether monetary reward enhances alpha suppression and working memory, and (iii) whether any performance benefits of neurofeedback-training would transfer to unrelated cognitive tasks. With the same experimental design, we conducted two studies with differing instructions given at the maintenance, yielding together 300 EEG recording sessions. In Study I, participants were engaged in a mental calculation task during maintenance. In Study II, they were instructed to visually rehearse the sample image. Results from Study I demonstrated a significant training and reward-anticipation effect on working memory accuracy and reaction times over 5 days. Neurofeedback and reward anticipation showed effects on theta suppression but not on alpha suppression. Moreover, a cognitive training effect was observed on beta suppression. Thus, neurofeedback-training of alpha was unrelated to working memory performance. Study II replicated the training and reward-anticipation effect on working memory but without any effects of neurofeedback-training on oscillations or working memory. Neither study showed transfer effects of either working memory or neurofeedback-training. A linear mixed-effect model analysis of neurofeedback-independent training-related improvement of working memory combining both studies showed that improved working memory performance was related to oscillatory changes over training days in the encoding and maintenance phases. Improvements in accuracy were related to increasing beta amplitude in reward trials over right parietal electrodes. Improvements in reaction times were related to increases in right parietal theta amplitude during encoding and increased right parietal and decreased left parietal beta amplitudes during maintenance. Thus, while our study provided no evidence that neurofeedback targeting alpha improved the efficacy of working memory training or evidence for transfer, it showed a relationship between training-related changes in parietal beta oscillations during encoding and improvements in accuracy. Right parietal beta oscillations could be an intervention target for improving working memory accuracy.

## Teaser

Does self-regulation of one’s own alpha frequency through neurofeedback improve working memory maintenance? In this study, Barbazzeni *et al.* found no evidence for neurofeedback-training effects based on a five days training regime. However, changes in right parietal low-beta power over training days were related to improved working memory accuracy.

## Graphical Abstract

**Graphical Abstract:**
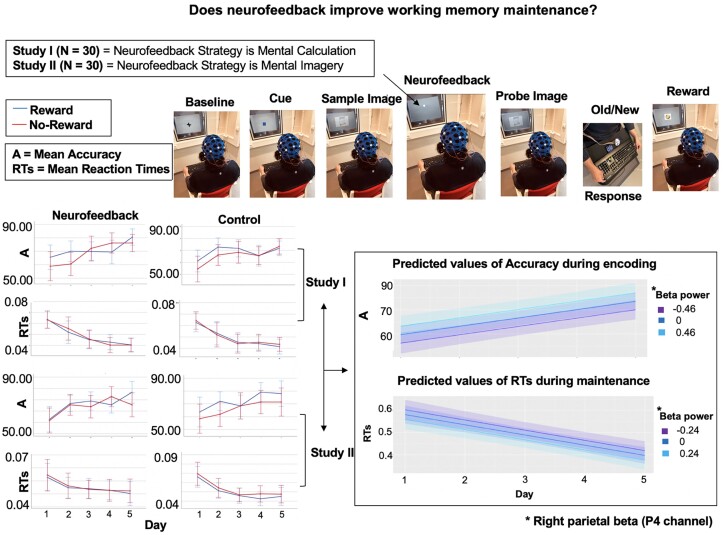

## Introduction

Working memory (WM) is the ability to hold information online beyond sensory stimulation.^1^ Maintaining information beyond sensory stimulation is believed to be supported by sustained neural activity patterns, including changes in neural oscillatory dynamics in the alpha frequency range.^2,3^ Changes in alpha oscillations have been associated with the suppression of irrelevant items, the selection of items to be maintained, and orienting attention in time, space, and context.^3^ Alpha oscillations change in response to stimulus/task demands through desynchronization (i.e. suppression) or synchronization.^4,5^ Alpha desynchronization has been related to anticipatory attention to a stimulus presentation, with stronger desynchronization being correlated with performance in discrimination tasks^6-8^ and cognitive effort.^9^

Motivation induced by the anticipation of monetary reward has been demonstrated to modulate the amplitude of sustained activity and the ability to inhibit distracting information during maintenance, thereby improving cognitive performance in a delayed match-to-sample task.^2^ Furthermore, the anticipation of monetary reward was found to be correlated with increased alpha suppression, indicating the influence of motivational factors on neural activity and cognitive performance.^10^ Regarding alpha suppression, several studies have investigated the role of neurofeedback training (NF) on alpha power to improve cognitive performance by enhancing neural activation.^7,8,11,12^ It remains unclear whether enhanced performance is correlated with increased^13-15^ or decreased^5,16-18^ alpha power.

In this study, we investigated whether NF-training of alpha suppression improves WM. Given the well-established effect of motivation by reward anticipation on WM, we investigated the interaction of NF-training with reward anticipation. Moreover, we also investigated the effect of cognitive training on theta and beta oscillations during maintenance due to their involvement in memory formation.^19-23^ Healthy young participants were trained across 5 days to increase alpha suppression during maintenance on the basis of real-time NF of their alpha oscillation amplitude or a control NF (CO) while performing a modified version of the delayed match-to-sample task (DMST)^24^ for visual objects in rewarded and unrewarded conditions. In half of the trials, participants were cued to anticipate monetary reward for correct DMST performance. We conducted two studies (Study I and Study II) that used the same experimental design but differed with respect to the instructions for the maintenance phase. In Study I, participants were instructed to perform a mental calculation (MC) unrelated to the DMST task while monitoring their alpha level. In Study II, participants were instructed to rehearse the sample objects (using mental imagery, MI) while monitoring their alpha level. In both studies, we also investigated whether NF-training facilitated the transfer of training DMST to unrelated cognitive tasks. Finally, by combining both studies, we investigated how WM training and ensuing behavioural improvements over 5 days were related to the modulation of specific neural oscillations during sample encoding and maintenance.

## Materials and methods

In Study I and Study II, we applied the same method.

### Participants

The study was approved by the Ethics Committee of the University Hospital Magdeburg (approval number 205/19) (Supplementary Material 1.1). The study protocol was explained in detail to all participants prior to signing the consent form, and withdrawal from the study was possible at any time. Participant confidentiality was maintained, and anonymous codes were assigned to participants. The exclusion criteria were neuropsychiatric disorders, history of epilepsy, chronic abuse (e.g. alcohol, drugs, analgesics, or other substances), participation in other clinical trials, and claustrophobia.

#### Study I

Thirty healthy young volunteers (13 males, 17 females; mean age = 25.04; SD = 2.428) took part in the experiment and were randomly assigned to two groups (NF versus CO). Participants were trained over five consecutive days in a double-blinded experiment. Fifteen participants (mean age = 24.74, SD = 2.743) received real-time NF of alpha power (NF group). Fifteen participants (mean age = 25.33, SD = 2.024) received control NF (CO-group).

#### Study II

Thirty healthy young volunteers (18 males, 12 females; mean age = 24.826; SD = 2.313) took part in the experiment and were randomly assigned to two groups (NF versus CO). Participants were trained over five consecutive days in a double-blinded experiment. Fifteen participants (mean age = 24.600, SD = 2.847) received a real-time NF of alpha power (NF group). Fifteen participants (mean age = 25.053, SD = 1.578) received a control NF (CO-group).

### Experimental design

Participants were randomly assigned to two groups (NF versus CO) based on the sealed envelope system.^25,26^ With eyes closed, the experimenter picked up one of two shuffled equal envelopes containing the type of treatment. The experimenter assistant was in charge of reading the content and assigning participants to the corresponding group. The experimenter and participants were blinded to the treatment for the entire duration of the training.

A familiarization session preceded five consecutive training days. Each training day consisted of two DMST blocks (15 min each). A DMST block consisted of 24 trials: 12 reward trials and 12 no-reward trials, each with six matching and six nonmatching probes (presented in random order). From a list of 878 computer-generated visual objects, stimuli were randomized across 10 DMST blocks that were presented to participants across 5 days of training. Stimuli were presented on a screen (size = 1680 × 1050 pixels, width = 47.5 cm, luminosity = 80, contrast = 80, visual angle = 10° 25′ 0.36′).

**Figure 1:**
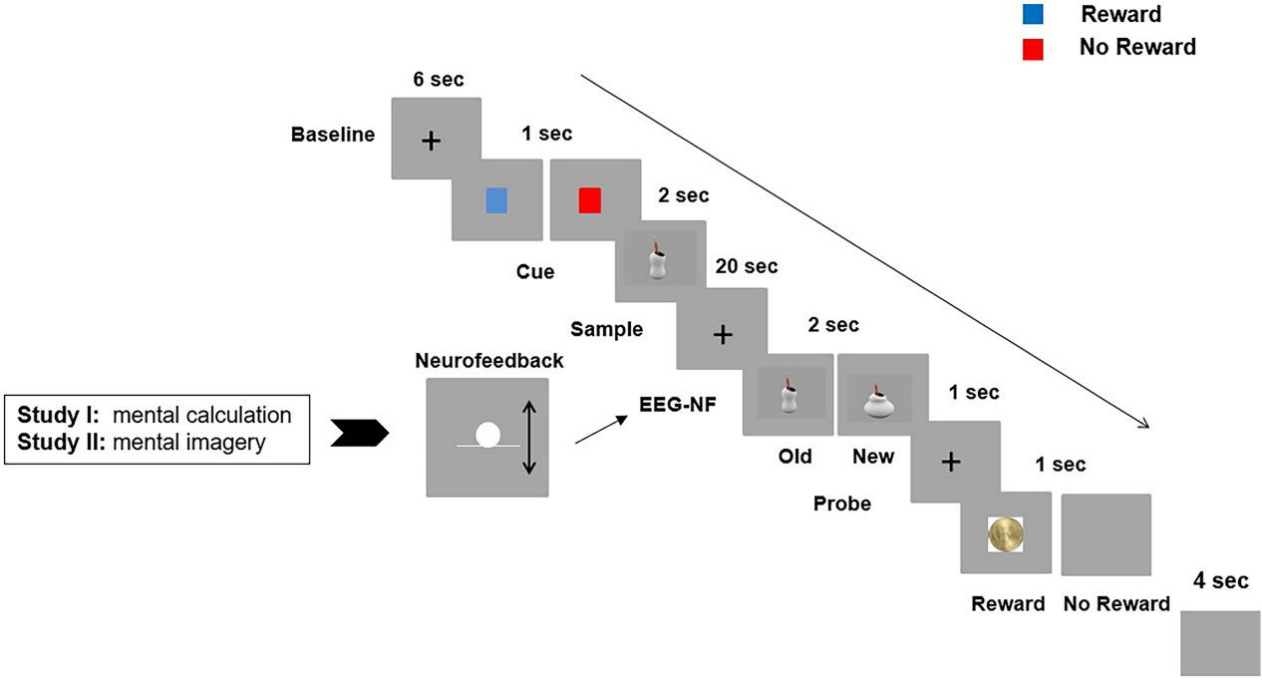
**Representation of one single trial in the monetary-rewarded DMST.** After a baseline represented as a fixation cross (6 s), a coloured cue was displayed (1 s). The cue could have been red or blue; the red cue predicted no monetary reward independent of the performance, whereas the blue cue predicted monetary reward depending on WM performance. Afterwards, the sample image was displayed for 2 s (i.e. a visual object). Then, the neurofeedback represented as a bouncing ball was given for 20 s, where participants were asked to control the movement of the ball towards the top of the screen. The movement of the ball towards the top was inversely proportional to the power of alpha in the NF group, whereas in the CO group, the movement of the ball was generated by random numbers. To enhance alpha power suppression, all participants in Study I were instructed to perform a mental calculation task, whereas all participants in Study II were instructed to perform a mental imagery task (to visually rehearse the sample image). Successively, the probe image was displayed (2 s), and participants were asked to make a choice between ‘old’ and ‘new’, where RTs were computed. Depending on the cue condition and participants’ performance, after a fixation cross of 1 s, a monetary reward was displayed (i.e. displayed as a 50-cent image) or not (i.e. grey screen) for 1 s. To switch from one trial to the other, a break of 4 s between trials was given (i.e. grey screen).

Before starting each DMST block, an individual's alpha range for each participant was estimated in a separate session. Then, the DMST started (Fig. 1). Each trial started with a baseline (6 s) wherein participants were asked to observe a fixation cross. Afterwards, a cue was presented (1 s), indicating whether correct performance in the upcoming trial would be rewarded or not. A blue square indicates a reward trial, while a red square indicates a no-reward trial. Participants were then presented with a trial-unique computer-generated visual object (i.e. sample image) for 2 s. This was followed by the maintenance period during which an NF signal was provided on the screen for 20 s. The trial concluded with a probe image presented for 2 s. Participants were required to judge whether the probe was a matching (‘old’) or a similar but new (‘new’) nonmatching version of the sample by pressing one of two buttons on a keyboard (counterbalanced for each DMST block within participants). In reward trials, correct responses were followed by a displayed reward (i.e. a 50-cent image), whereas incorrect responses were followed by a blank grey screen. In no-reward trials, correct and incorrect responses were followed by a blank grey screen. We assessed task performance based on participants’ accuracy and RTs during the recollection process. The task instructions were displayed in two possible languages (English or German), depending on the participants’ choice. Participants received a daily compensation of 7 euros for their participation. However, depending on their daily performance, they had the opportunity to gain an additional monetary reward (i.e. up to 2 euros) but only in reward trials.

To enhance alpha suppression during maintenance, we instructed participants to implement a mental strategy. In Study I, participants were instructed to perform an MC task, which consisted of performing a mental subtraction (e.g. starting from 700, subtract each time −7). In Study II, they were instructed to visually rehearse the sample image of the current trial (MI). In addition to the maintenance instructions, participants were instructed to suppress alpha power based on the displayed NF or CO-signal. In the NF group, participants were trained to increase alpha suppression on the basis of a real-time NF and related to their individual alpha range, whereas the CO group received the same instructions and procedures but was trained based on a control signal generated by random numbers. In both groups, the feedback signal was displayed as a white ball moving in real time. In NF, the movement of the ball was proportional to the relative power of alpha. A movement closer to the top of the screen indicated higher alpha suppression, whereas a movement closer to the baseline line indicated decreased alpha suppression. In CO, the movement of the ball was equal, although it was generated by random numbers.

The presentation and timing of all DMST stimuli were controlled using PsychoPy^27^ in Python.

In reference to signal detection theory,^28^ we computed the accuracy in the DMST as the difference between hit rates (H) and false alarms (FAs). In the case in which the sample was ‘old’ and the probe would have been ‘old’, Hit indicated a correct performance, whereas in the case in which the sample was ‘new’ but the probe would have been identified as ‘old’, FA indicated an incorrect performance.

### EEG recording and neurofeedback

We recorded alpha oscillations using one set of OpenBCI^29^ Gold Cup Electrodes. A Multi-Cap Cup^30^ and OneStep Cleargel^31^ were used to adhere the electrodes. For the NF-training, we used seven electrodes placed in accordance with the 10–20 International System and one in ear-electrode. To reduce the influence of eye movements artefacts from frontal areas, the locations were TP8, C4, in ear-EEG, C3, P4, Pz, P3, and TP7. The right mastoid (TP10) was used as a reference for all electrodes, the left mastoid (TP9) as a noise-cancelling ground electrode. Impedance was kept below 5 kΩ for all electrodes.

In the NF and CO groups, we sampled the EEG by using all eight channels on the OpenBCI Cyton Board.^29^ As part of the training protocol before the start of each DMST block and for each training day, we identified an individual’s alpha range based on which the individual training was performed. The individual’s alpha range was estimated during an eyes-open baseline block of 2 min, where participants were observing a displayed fixation cross on the computer screen. Three possible individual ranges (6–10 Hz, 7–11 Hz, or 8–12 Hz) were estimated by applying a fast Fourier transform (FFT). To generate the NF signal, we filtered the raw signal based on the individual’s alpha range (with no restrictions on the amplitude changes of neighbouring bands). Then, we segmented the signal into sliding windows of 0.25 s by applying the FFT to each window. Successively, we normalized the signal trial-by-trial with its respective baseline (6 s). Considering signal acquisition and preprocessing time, the total delay between EEG-signal and neurofeedback was 0.49 s. The Lab Streaming Layer ^32^ in Python provided the online processing of the NF signal.

### EEG preprocessing and spectral analysis

The EEG activity from the OpenBCI system was recorded in volts (V). We used the EEGLAB^33^ version eeglab_15x toolbox in MATLAB 2018b^34^ to import raw data, events, and channel locations and to interpolate bad channels. We used MNE software version 0.18 in Python^35,36^ to load and epoch the preprocessed data. We epoched the raw signal for reward and no-reward trials within each DMST block. Each epoch included five events from the baseline to the end of the probe image presentation (i.e. each epoch length was 31 s). We used the ‘Autoreject API’ library in Python^37,38^ to remove artefacts and to reject bad trials, and an individual threshold was set to reject trials with artefacts. Moreover, we further analysed the signal in MATLAB 2018b^34^ with Morlet Wavelets time-frequency analysis and filtered the signal in a range of 2–20 Hz with a resolution of 0.5 Hz. To investigate the maintenance period, for averaged epochs and alpha (7–13 Hz), theta (4–7 Hz), and low-beta (13–20 Hz) frequencies, the analysed signal corresponding to the NF event was normalized using the decibel log-ratio formula (10*log_10_(Activity\Baseline))^39^ relative to the baseline event (only 5 s was considered for the computation).

In studies I and II, we computed this analysis for each subject, channel location, reward condition, and training day. Then, a grand average between participants was performed to investigate the power of oscillations for each training day, reward condition, channel location, and group.

### Transfer effect tasks

To evaluate transfer effects, participants were instructed to perform one block of a Mnemonic Similarity Test (MST) and one block of a Stroop task before Day 1 and after Day 5 of the training. These tasks were displayed in two possible languages (English or German).

An adapted version of the MST^40^ was used. The MST involved an incidental study phase where participants were presented 128 images of visual objects for 2 s on the computer screen. Participants had to judge each object as indoor or outdoor by pressing a button on a keyboard. Afterwards, in a test phase, participants were asked to classify visual objects presented for 2 s as ‘old’ (repetitions), ‘similar’ (lures), or ‘new’ (foils) by pressing different buttons. ‘Old’ objects were already presented in the study phase, ‘similar’ objects were similar but different from objects already presented, and ‘new’ were objects never presented. The test phase included 192 trials: 64 repetitions, 64 lures, and 64 foils.

During the Stroop task,^41^ words were displayed for 2 s with different colours and meanings. Word colours and word meanings were congruent or not. For each displayed word, participants were asked to consider the word colour (phase 1) or the word meaning (phase 2). The task consisted of 280 trials equally divided for each phase. The choice of colours was ‘green’, ‘blue’, ‘yellow’, and ‘red’.

The presentation and timing of all stimuli in the MST and Stroop task were controlled using Presentation® software^42^ version 18.0 and Psychopy^27^ in Python, respectively. We computed the performance of the MST and Stroop tasks based on accuracy.

### Questionnaire post neurofeedback training

At the conclusion of the experiment, participants were given a questionnaire (English or German). Twelve questions (Supplementary Appendix A) were asked to generally investigate their opinion and feelings about the experiment, their memory performance estimation across the training, task difficulty, and level of engagement. The questionnaire responses were evaluated based on a customized 6-point Likert scale, where 1 = ‘absolutely not good’, 2 = ‘not good’, 3 = ‘slightly not good’, 4 = slightly good’, 5 = ‘good’, 6 = ‘very good’, or from 1 = ‘absolutely not difficult’, 2 = ‘not difficult’, 3 = slightly not difficult’, 4 = ‘slightly difficult’, 5 = ‘difficult’, 6 = ‘very difficult’, and on a ‘yes’ and ‘no’ scale.

### Statistical analysis

#### Study I and Study II—separate analyses

The DMST accuracy and RTs were investigated with two-way mixed repeated-measures ANOVAs. Within-subjects factors were ‘day’ (5 levels: Day 1, Day 2, Day 3, Day 4, and Day 5) and ‘reward condition’ (2 levels: reward and no-reward). The between-subjects factor was ‘group’ (NF versus CO) with ‘gender’ as a covariate on the mean distribution. However, ‘gender’ was removed from the accuracy analysis because it was found to significantly affect the results. Nevertheless, considering our sample from the same population (young healthy volunteers) and the factor gender unrelated to the hypotheses under investigation, this covariate was removed. The performance of the MST and Stroop tasks was investigated with nonparametric statistics (Supplementary Material 1.2). The Mann–Whitney U-test (or independent samples *t*-test) was used to perform this between-subject analysis. The parameter ‘U’ indicates the test statistic, whereas ‘N’ indicates the mean rank. Moreover, we analysed the questionnaire based on frequencies that were computed based on the median distribution, where the corresponding percentage value of the most frequent answer was considered.

To statistically investigate the results from the time-frequency analyses, we applied three-way mixed repeated-measures ANOVAs. The within-subjects factors were ‘day’, ‘reward condition’, and ‘channel’ (3 levels: P4, Pz, and P3). The between-subjects factor was ‘group’ with ‘gender’ as a covariate. Only for this analysis, due to an odd channel electrode occurring during the measurement, and therefore generating data deviating from the normal distribution, was one participant (from the CO group, in Study II) removed as an outlier. Any corrected ANOVA results were made with Greenhouse–Geisser. Unless otherwise stated, the Huynh–Feldt correction was used.

All statistical analyses were executed using SPSS software version 26.0.^43^

#### Combined analysis across Study I and Study II

We further investigated how WM improvements over 5 days were related to specific oscillatory changes measured during sample encoding and maintenance.

Participants in Study I and Study II were considered one sample (*n* = 60, female = 29, male = 31, mean age = 24.95, SD = 2.389). We removed one participant (from the CO group, in Study II) as an outlier; therefore, the analysed sample consisted of 59 participants.

We considered the power for theta, alpha, and low-beta during encoding and maintenance for parietal channels P4 and P3. To investigate neural oscillations at encoding (sample image presentation), we considered the first 1000 ms. Previous electrophysiological studies demonstrated that encoding occurs rapidly,^44^ suggesting that beta^45^ and alpha^46,47^ oscillations can influence memory encoding within 500 ms after stimulus onset, whereas the influence of theta oscillations^47-49^ was likely to extend over a longer interval comprising at least the first 1000 ms after stimulus onset. Therefore, we divided the encoding period into two 500-ms time windows (S1 and S2): S1 from 0 to 500 ms and S2 from 500 to 1000 ms. The analysed signal corresponding to each time window was baseline normalized (the last 500 ms of the baseline period before stimulus onset was considered).

To assess the relationship between oscillatory changes and WM across 5 days, we conducted an exploratory analysis with a linear mixed-effect model (LMM)^50^ in MATLAB 2018b.^34^ We built models based on restricted maximum likelihood (REML) identifying accuracy and RTs as target variables [for further model specifications, see (Supplementary Material 1.3) ]. Alternative hypothesis models (H_1_) were built under the hypothesis that even when considering the variability in performance across 5 days and participants, WM improvements were not random but related to specific experimental variables observed during encoding or maintenance. In contrast, a null hypothesis model (H_0_) was built under the hypothesis that WM improvements were explained only by the random variability in performance across 5 days. Thus, we compared model adequacy with the maximum likelihood (ML) ratio test to investigate the statistical significance of H_1_ over H_0_. First, we investigated the main effects. Second, interaction effects were assessed in a *post hoc* analysis.

### Data availability statement

The data are not publicly available due to restrictions (the conditions of our ethics approval do not permit public archiving of pseudonymized study data). Access can be granted to individuals in accordance with ethical procedures governing the reuse of sensitive data.

## Results

### Study I

Additional results of Study I are in Supplementary Material 2.

#### Delayed match-to-sample task

**Figure 2:**
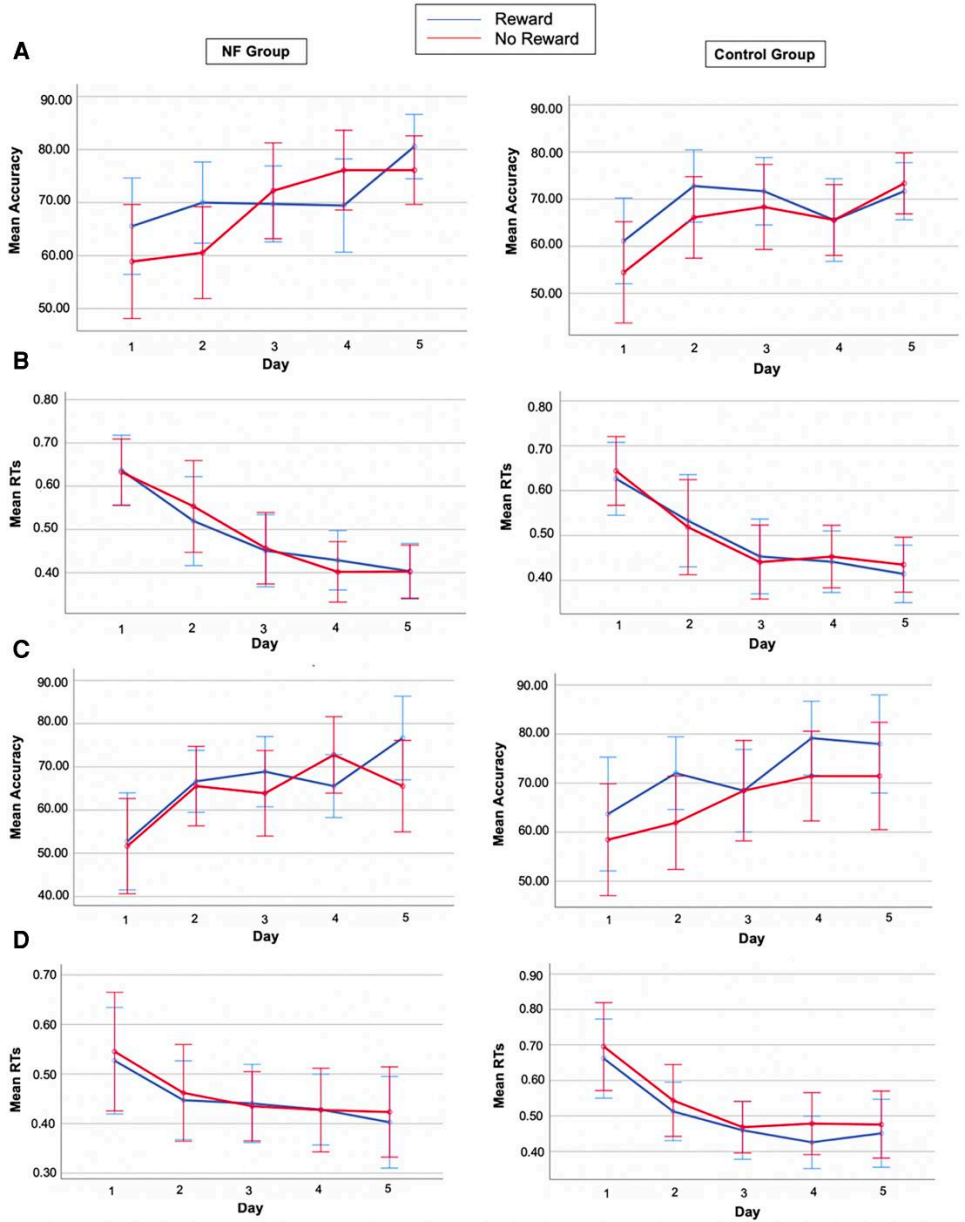
**Accuracy and reaction time results in Study I and Study II. A** Accuracy is represented for the NF group (left) and for the CO group (right) in Study I. In both figures, training days are represented on the *x*-axis, and mean accuracy is represented on the *y*-axis. Blue lines denote behavioural data from the reward condition. Red lines denote behavioural data from the no-reward condition. Accuracy improved across 5 days (*F*_(4,112)_ = 8.302, *P* = 0.000, *η*_p_^2^ = 0.229). **B** RTs are represented for the NF group (left) and for the CO group (right) in Study I. In both figures, training days are represented on the *x*-axis, and mean RTs are represented on the *y*-axis. Blue lines denote behavioural data from the reward condition. Red lines denote behavioural data from the no-reward condition. RTs improved across 5 days (*F*_(2.800,75.601)_ = 3.318, *P* = 0.027, *η*_p_^2^ = 0.109) with a significant reward-anticipation effect (*F*_(1,27)_ = 8.344, *P* = 0.008, *η*_p_^2^ = 0.236). **C** Accuracy is represented for the NF group (left) and for the CO group (right) in Study II. In both figures, training days are represented on the *x*-axis and mean accuracy on the *y*-axis. Blue lines denote behavioural data from the reward condition. Red lines denote behavioural data from the no-reward condition. Accuracy improved across 5 days (*F*_(4,112)_ = 8.991, *P* = 0.000, *η*_p_^2^ = 0.243) with a significant reward-anticipation effect (*F*_(1,28_) = 5.313, *P* = 0.029, *η*_p_^2^ = 0.159). **D** RTs are represented for the NF group (left) and for the CO group (right) in Study II. In both figures, training days are represented on the *x*-axis and mean RTs on the *y*-axis. Blue lines denote behavioural data from the reward condition. Red lines denote behavioural data from the no-reward condition. RTs improved across 5 days (*F*_(2.009,54.241)_ = 3.183, *P* = 0.049, *η*_p_^2^ = 0.105) with a significant reward-anticipation effect (*F*_(1,27)_ = 4.458, *P* = 0.044, *η*_p_^2^ = 0.142 Red lines denote behavioural data from the no-reward condition. Error bars are 95% confidence intervals (CI) and factor gender as covariates.

We found improved DMST accuracy (Fig. 2A) across 5 days (main effect of day: *F*_(4,112)_ = 8.302, *P* = 0.000, *η*_p_^2^ = 0.229), although it was not influenced by reward anticipation (main effect of reward: *F*_(1,28)_ = 2.557, *P* = 0.121, *η*_p_^2^ = 0.084). No significant differences in performance between groups (main effect of group: *F*_(1,28)_ = 1.101, *P* = 0.303, *η*_p_^2^ = 0.038) were observed across the 5 days (interaction day*group: *F*_(4,112)_ = 1.374, *P* = 0.247, *η*_p_^2^ = 0.047) or interactions between NF-training and reward anticipation (interaction day*reward*group: *F*_(4,112)_ = 0.606, *P* = 0.659, *η*_p_^2^ = 0.021) effects on accuracy. Furthermore, DMST RTs (Fig. 2B) improved across 5 days (main effect of day: *F*_(2.800,75.601)_ = 3.318, *P* = 0.027, *η*_p_^2^ = 0.109) with a significant reward-anticipation effect (main effect of reward: *F*_(1,27)_ = 8.344, *P* = 0.008, *η*_p_^2^ = 0.236). No significant differences in performance between groups (main effect of group: *F*_(1,27)_ = 0.034, *P* = 0.856, *η*_p_^2^ = 0.001) were observed across the 5 days (interaction day*group: *F*_(2.800,75.601)_ = 0.232, *P* = 0.861, *η*_p_^2^ = 0.009) or interactions between NF-training and reward anticipation (interaction day*reward*group: *F*_(4,108)_ = 1.486, *P* = 0.212, *η*_p_^2^ = 0.052) effects on RTs.

#### Alpha power (7–13 Hz)

**Figure 3:**
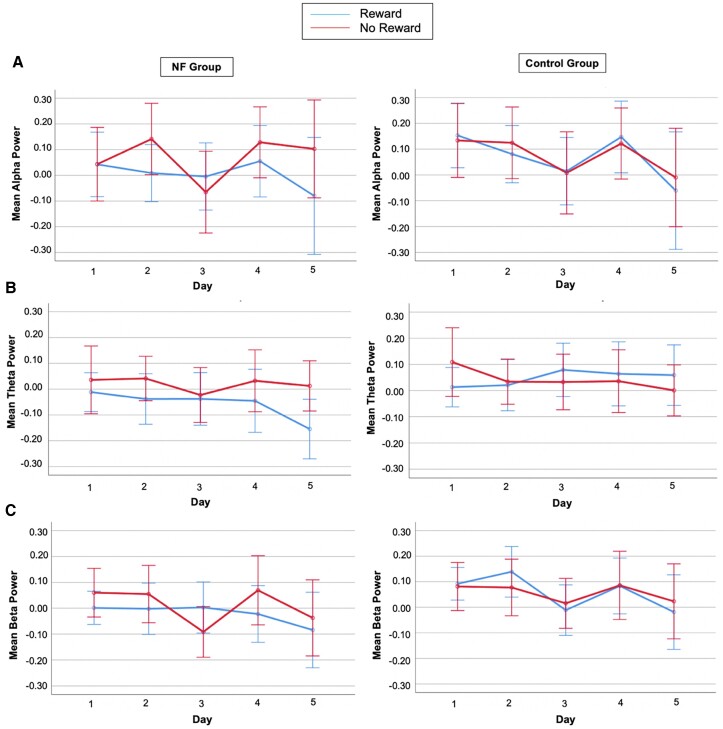
**Mean alpha, theta, and low-beta power results in Study I. A** Channels P3, Pz, and P4 were considered for the time-frequency analysis, and the displayed signal is the grand average across these channels. Alpha power is represented for the NF group (left) and for the CO group (right). In both figures, training days are represented on the *x*-axis and mean alpha power on the *y*-axis. Blue lines denote time-frequency data from the reward condition. Red lines denote time-frequency data from the no-reward condition. **B** Channels P3, Pz, and P4 have been considered for the time-frequency analysis, and the displayed signal is the grand average across these channels. Theta power is represented for the NF group (left) and for the CO group (right). In both figures, training days are represented on the *x*-axis and mean theta power on the *y*-axis. Blue lines denote time-frequency data from the reward condition. Red lines denote time-frequency data from the no-reward condition. Although no significant differences were found in the relative theta power across 5 days, groups, or reward anticipation, the interaction between reward anticipation and the type of NF or CO training (*F*_(1,27)_ = 4.881, *P* = 0.036, *η*_p_^2^ = 0.153) was significant. Furthermore, when considering Day 1 and Day 5, the interaction between NF training and reward anticipation (*F*_(1,27)_ = 5.056, *P* = 0.033, *η*_p_^2^ = 0.158) was also significant. **C** Channels P3, Pz, and P4 were considered for the time-frequency analysis, and the displayed signal is the grand average across these channels. Beta power is represented for the NF group (left) and for the CO group (right). In both figures, training days are represented on the *x*-axis and mean beta power on the *y*-axis. Blue lines denote time-frequency data from the reward condition. Red lines denote time-frequency data from the no-reward condition. Error bars are 95% confidence intervals (CI) and factor gender as covariates. A significant decrease in relative low-beta power was found across 5 days (*F*_(2.581,69.677)_ = 3.775, *P* = 0.019, *η*_p_^2^ = 0.123).

We found no significant differences in the relative alpha power across 5 days (main effect day: *F*_(2.543,68.672)_ = 1.480, *P* = 0.232, *η*_p_^2^ = 0.052), groups (main effect of group: *F*_(1,27)_ = 0.467, *P* = 0.500, *η*_p_^2^ = 0.017), or reward anticipation (main effect of reward: *F*_(1,27)_ = 1.031, *P* = 0.319, *η*_p_^2^ = 0.037). No significant interaction effects between NF-training and reward anticipation across training (day*reward*group: *F*_(2.617,70.668)_ = 0.390, *P* = 0.734, *η*_p_^2^ = 0.014) were found on alpha suppression. However, when investigating the power of alpha between Day 1 and Day 5, the day*reward*channel interaction (*F*_(2,54)_ = 4.743, *P* = 0.013, *η*_p_^2^ = 0.149) was significant (Fig. 3A).

#### *Post hoc* analyses

**Figure 4:**
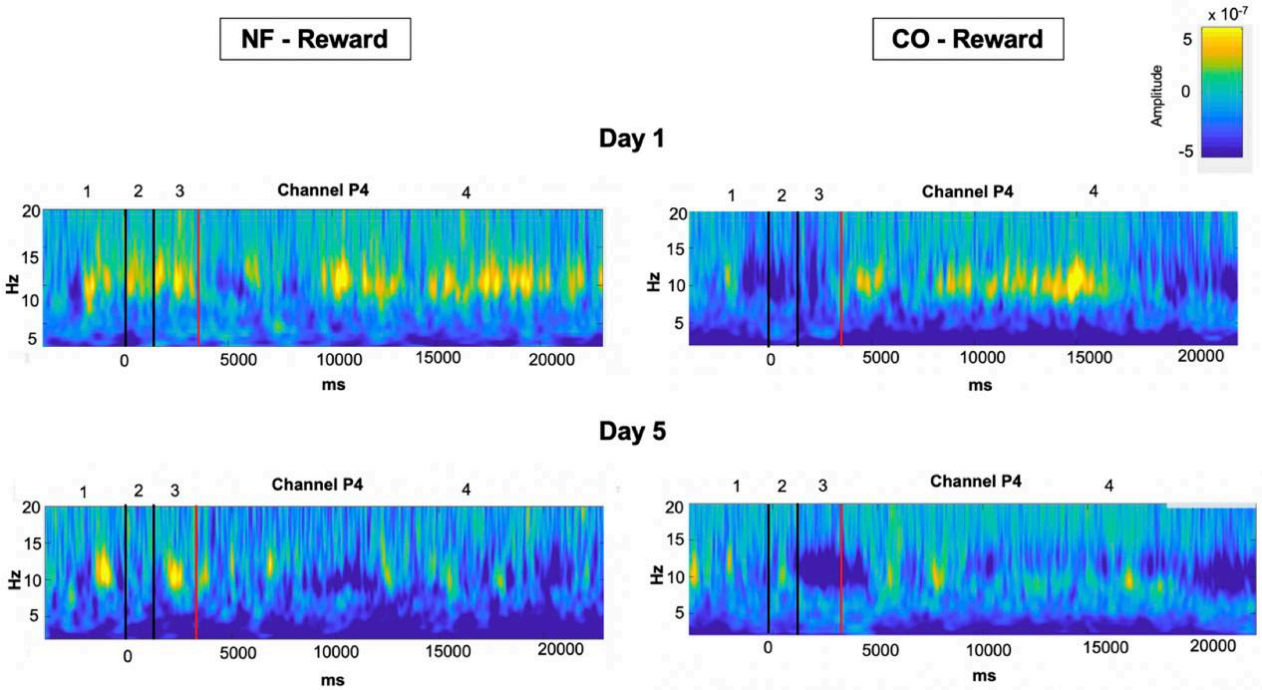
**Study I: for illustration purposes, graphical representations of the power spectrum from the time-frequency analysis for both groups in reward condition.** The power spectrum is represented for channel P4 for the NF (left) and CO groups (right). Only Day 1 (top) and Day 5 (bottom) are represented for the reward condition. On the *x*-axis, the time is represented from −5000 to 23 000 ms. This time period includes baseline, cue presentation, sample image, and neurofeedback period, where 0 ms represents the presentation of the cue predicting reward. The red line indicates the beginning of the neurofeedback period. In addition, black lines that separate the events and numbers clarify which events appeared for this event time period. Thus, number 1 indicated the baseline, number 2 the presentation of the cue predicting reward, number 3 the sample image presentation, and number 4 the neurofeedback period. On the *y*-axis, frequencies from 2 to 20 Hz with a resolution of 0.5 Hz are represented.

Visual analysis of time-frequency plots (Fig. 4) shows that on Day 1 in reward trials and after encoding, the NF group showed strong alpha suppression during the first third of the maintenance phase when compared to the CO group. This suggested that the NF effect on alpha suppression could have been short-lived and did not span the entire maintenance period. Thus, we performed a paired *t*-test *post hoc* analysis to investigate whether alpha suppression (in P4, on Day 1, reward trials) was different between groups during the first seven seconds of the maintenance phase. Although the mean alpha power was higher in the CO group than in the NF group [M = 0.24049, 95% CI (−0.11241.59339)], the difference was not significant (*t*_14_ = 1.462, *P* = 0.166).

#### Theta power (4–7 Hz)

We found no significant differences in the relative theta power across 5 days (main effect of day: *F*_(4,108)_ = 2.271, *P* = 0.066, *η*_p_^2^ = 0.078), groups (main effect of group: *F*_(1,27)_ = 2.459, *P* = 0.129, *η*_p_^2^ = 0.083), or reward anticipation (main effect of reward: *F*_(1,27)_ = 0.015, *P* = 0.904, *η*_p_^2^ = 0.001). However, the interaction between reward anticipation and the type of NF or CO training (reward*group: *F*_(1,27)_ = 4.881, *P* = 0.036, *η*_p_^2^ = 0.153) was significant. Pairwise comparisons indicated that theta suppression was generally higher in the NF group than in the CO group and was higher in reward (MD = −0.105, SEM = 0.044, *P* = 0.025) compared to no-reward (MD = −0.023, SEM = 0.045, *P* = 0.621) trials. Moreover, when considering Day 1 and Day 5, the interaction between NF-training and reward anticipation (day*reward*group: *F*_(1,27)_ = 5.056, *P* = 0.033, *η*_p_^2^ = 0.158) was significant. Pairwise comparisons showed higher theta suppression on Day 5 in NF (compared to CO) for reward trials (MD = −0.214, SEM = 0.080, *P* = 0.012) but not no-reward trials (MD = 0.012, SEM = 0.067, *P* = 0.860) (Fig. 3B).

#### Low-beta power (13–20 Hz)

We found a significant decrease in relative low-beta power across 5 days (main effect of day: *F*_(2.581,69.677)_ = 3.775, *P* = 0.019, *η*_p_^2^ = 0.123). Pairwise comparisons showed decreased power on Day 5 compared to Day 4 (MD = −0.083, SEM = 0.040, *P* = 0.046) and Day 2 (MD = −0.096, SEM = 0.047, *P* = 0.05). However, beta power did not differ between groups (main effect of group: *F*_(1,27)_ = 1.710, *P* = 0.202, *η*_p_^2^ = 0.060) and reward conditions (main effect of reward: *F*_(1,27)_ = 1.226, *P* = 0.270, *η*_p_^2^ = 0.045). There was no significant interaction between NF-training and reward anticipation across training (day*reward*group: *F*_(4,108)_ = 1.354, *P* = 0.255, *η*_p_^2^ = 0.048). Furthermore, when considering Day 1 and Day 5, neither the main effects nor interactions were significant (Fig. 3C).

#### Transfer effect tasks

No significant differences were found on the MST (Day 1: *U*(*N*_NF_ = 18.53, *N*_CO_ = 12.47) = 67.000, *z* = −1.888, *P* = 0.061; Day 5: *U*(*N*_NF_ = 17.83, *N*_CO_ = 13.17) = 77.500, *z* = −1.452, *P* = 0.148), or the Stroop task (Day 1: *U*(*N*_NF_ = 19.27, *N*_CO_ = 11.73) = 56.000, *z* = −2.347, *P* = 0.019; Day 5: *U*(*N*_NF_ = 18.90, *N*_CO_ = 12.10) = 61.500, *z* = −2.122, *P* = 0.033). No NF-training effects on unrelated cognitive tasks were found.

#### Questionnaire

Questionnaire results are reported in Supplementary Material 2.6.

### Study II

Additional results of Study II are provided in Supplementary Material 3.

#### Delayed match-to-sample task

We found improved DMST accuracy (Fig. 2C) across 5 days (main effect of day: *F*_(4,112)_ = 8.991, *P* = 0.000, *η*_p_^2^ = 0.243) with a significant reward-anticipation effect (main effect of reward: *F*_(1,28)_ = 5.313, *P* = 0.029, *η*_p_^2^ = 0.159). Pairwise comparisons showed higher accuracy in reward (MD = 4.156, SEM = 1.803, *P* = 0.029) than in no-reward trials. No significant differences in performance between groups (main effect of group: *F*_(1,28)_ = 2.869, *P* = 0.101, *η*_p_^2^ = 0.093) were observed across 5 days (interaction day*group: *F*_(4,112)_ = 0.525, *P* = 0.717, *η*_p_^2^ = 0.018) or interactions between NF-training and reward anticipation (interaction day*reward*group: *F*_(4,112)_ = 0.993, *P* = 0.414, *η*_p_^2^ = 0.034) on accuracy. Furthermore, DMST RTs (Fig. 2D) improved across 5 days (main effect of day: *F*_(2.009,54.241)_ = 3.183, *P* = 0.049, *η*_p_^2^ = 0.105) with a significant reward-anticipation effect (main effect of reward: *F*_(1,27)_ = 4.458, *P* = 0.044, *η*_p_^2^ = 0.142). Pairwise comparisons showed faster RTs in reward trials (MD = −0.018, SEM = 0.006, *P* = 0.005) than in no-reward trials. No significant differences in performance between groups (main effect of group: *F*_(1,27)_ = 1.823, *P* = 0.188, *η*_p_^2^ = 0.063) were observed across 5 days (interaction day*group: *F*_(2.009,54.241)_ = 1.419, *P* = 0.251, *η*_p_^2^ = 0.050) or interactions between NF-training and reward anticipation (interaction day*reward*group: *F*_(2.831,76.445)_ = 0.722, *P* = 0.535, *η*_p_^2^ = 0.026) on RTs.

#### Alpha power (7–13 Hz)

**Figure 5:**
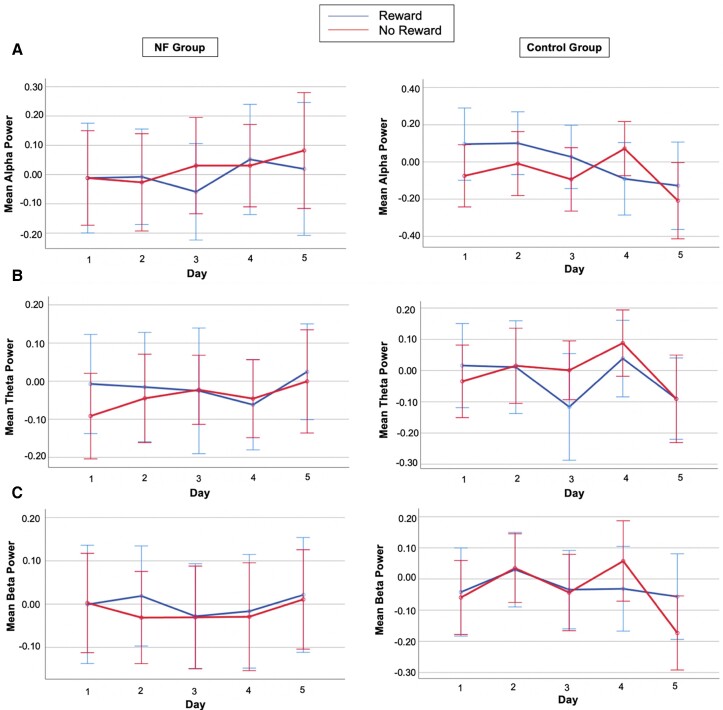
**Mean alpha, theta, and low-beta power results in Study II. A** Channels P3, Pz, and P4 were considered for the time-frequency analysis, and the displayed signal is the grand average across these channels. Alpha power is represented for the NF group (left) and for the CO group (right). In both figures, training days are represented on the *x*-axis and mean alpha power on the *y*-axis. Blue lines denote time-frequency data from the reward condition. Red lines denote time-frequency data from the no-reward condition. **B** Channels P3, Pz, and P4 have been considered for the time-frequency analysis, and the displayed signal is the grand average across these channels. Theta power is represented for the NF group (left) and for the CO group (right). In both figures, training days are represented on the *x*-axis and mean theta power on the *y*-axis. Blue lines denote time-frequency data from the reward condition. Red lines denote time-frequency data from the no-reward condition. **C** Channels P3, Pz, and P4 were considered for the time-frequency analysis, and the displayed signal is the grand average across these channels. Beta power is represented for the NF group (left) and for the CO group (right). In both figures, training days are represented on the *x*-axis and mean beta power on the *y*-axis. Blue lines denote time-frequency data from the reward condition. Red lines denote time-frequency data from the no-reward condition. Error bars are 95% confidence intervals (CI) and factor gender as covariates.

We found no significant differences in the relative alpha power across 5 days (main effect day: *F*_(2.908,75.602)_ = 1.617, *P* = 0.194, *η*_p_^2^ = 0.059), groups (main effect of group: *F*_(1,26)_ = 0.251, *P* = 0.621, *η*_p_^2^ = 0.010), or reward anticipation (main effect of reward: *F*_(1,26)_ = 0.339, *P* = 0.565 *η*_p_^2^ = 0.013). No significant interaction effects between NF-training and reward anticipation (day*reward*group: *F*_(3.148,81.860)_ = 1.479, *P* = 0.225, *η*_p_^2^ = 0.054) on alpha suppression were found. Furthermore, considering Day 1 and Day 5, neither the main effects nor interactions were significant (Fig. 5A).

#### Theta power (4–7 Hz)

We found no significant differences in the relative theta power across 5 days (main effect of day: *F*_(4,104)_ = 0.719, *P* = 0.581, *η*_p_^2^ = 0.027), groups (main effect of group: *F*_(1,26)_ = 0.105, *P* = 0.748, *η*_p_^2^ = 0.004), or reward anticipation (main effect of reward: *F*_(1,26)_ = 0.080, *P* = 0.779, *η*_p_^2^ = 0.003). No significant interactions between NF-training and reward anticipation across 5 days (day*reward*group: *F*_(4,104)_ = 0.120, *P* = 0.975, *η*_p_^2^ = 0.005) were found on theta. Furthermore, considering Day 1 and Day 5, neither the main effects nor interactions were significant (Fig. 5B).

#### Low-beta power (13–20 Hz)

We found no significant differences in the relative low-beta power across 5 days (main effect of day: *F*_(2.903,75.481)_ = 0.678, *P* = 0.563, *η*_p_^2^ = 0.025), groups (main effect of group: *F*_(1,26)_ = 0.249, *P* = 0.622, *η*_p_^2^ = 0.009), or reward anticipation (main effect of reward: *F*_(1,26)_ = 2.790, *P* = 0.107, *η*_p_^2^ = 0.097). No significant interaction effects between the NF-training and reward anticipation across 5 days (day*reward*group: *F*_(2.720,70.731)_ = 0.469, *P* = 0.686, *η*_p_^2^ = 0.018) were found on beta power. Furthermore, considering Day 1 and Day 5, neither the main effects nor interactions were significant (Fig. 5C).

**Figure 6:**
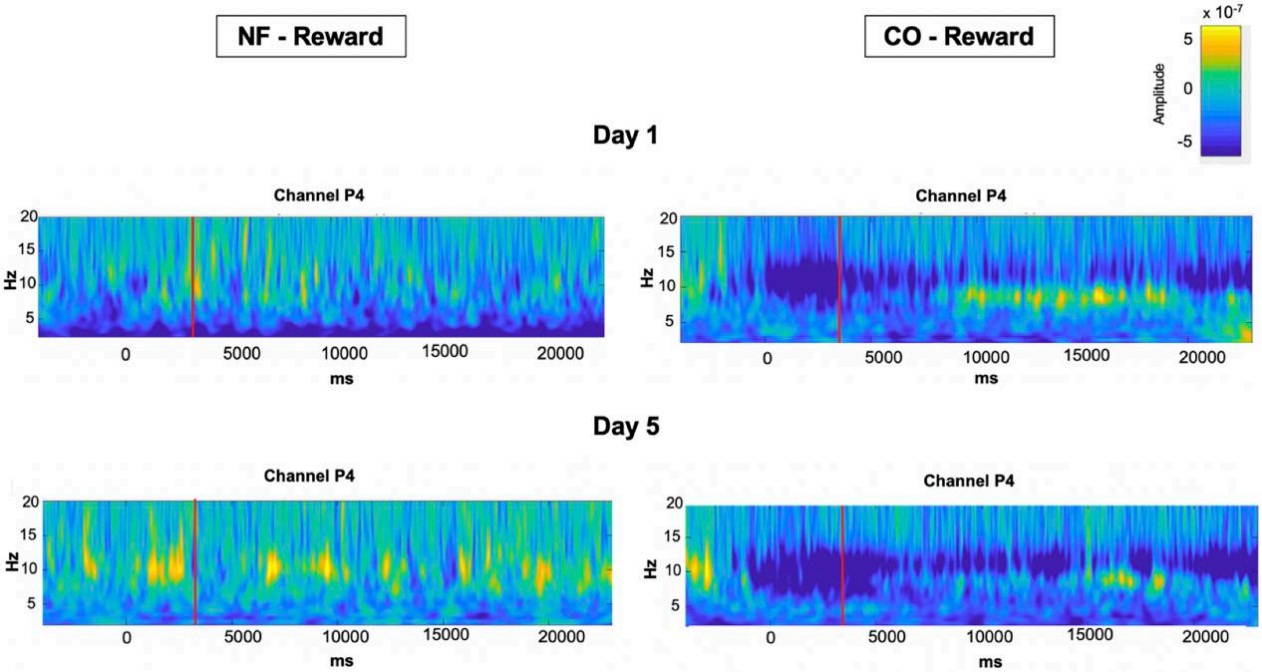
**Study II: for illustration purposes, graphical representations of the power spectrum from the time-frequency analysis for both groups in reward condition.** The power spectrum is represented for channel P4 for the NF (left) and CO groups (right). Only Day 1 (top) and Day 5 (bottom) are represented for the reward condition. On the *x*-axis, the time is represented from −5000 to 23 000 ms. This time period includes baseline, cue presentation, sample image, and neurofeedback period, where 0 ms represents the presentation of the cue predicting reward. The red line indicates the beginning of the neurofeedback period. On the *y*-axis, frequencies from 2 to 20 Hz with a resolution of 0.5 Hz are represented.

Figure 6 shows the power spectrum of Study II.

#### Transfer effect tasks

No significant differences were found on the MST (Day 1: *U*(*N*_NF_ = 14.73, *N*_CO_ = 16.27) = 101.000, *z* = −0.477, *P* = 0.653; Day 5: *U*(*N*_NF_ = 15.57, *N*_CO_ = 15.43) = 111.500, *z* = −0.041, *P* = 0.967) or the Stroop task (Day 1: *U*(*N*_NF_ = 14.80, *N*_CO_ = 16.20) = 102.000, *z* = −0.437, *P* = 0.683; Day 5: *U*(*N*_NF_ = 14.50, *N*_CO_ = 16.50) = 97.500, *z* = −0.623, *P* = 0.539). No NF-training effects on unrelated cognitive tasks were found.

#### Questionnaire

Questionnaire results are reported in Supplementary Material 3.6.

### Combined analysis across studies

Table 1 reports the results from encoding, whereas Table 2 reports the results from maintenance.

**Table 1:** Model specification and fixed effect coefficients with related statistics

Model specification: Linear mixed-effect model for accuracy during encoding				
Fixed effect coefficients	Estimate (coefficients)	*P* value	Lower (95% CI)	Upper (95% CI)
‘Intercept’	56.137	0	51.747	60.526
‘Group_NF’^a^	−0.93638	0.63793	−4.8425	2.9697
‘Reward’^b^	**3.3522**	0.01034**	**0.79302**	**5.9115**
‘Day’^c^	**3.4545**	4.1878e−12***	**2.4963**	**4.4126**
‘P4_S1_Alpha’^d^	−0.29695	0.89029	−4.5234	3.9295
‘P3_S1_Alpha’^e^	2.2286	0.33152	−2.2753	6.7325
‘P4_S2_Alpha’^f^	−2.4383	0.25215	−6.6163	1.7396
‘P3_S2_Alpha’^g^	0.17891	0.93611	−4.2028	4.5606
‘P4_S1_Theta’^h^	−2.7649	0.19333	−6.9349	1.405
‘P3_S1_Theta’^i^	1.3674	0.51424	−2.7477	5.4825
‘P4_S2_Theta’^j^	3.765	0.08581	−0.53213	8.0621
‘P3_S2_Theta’^k^	−3.5677	0.07927	−7.5537	0.41822
‘P4_S1_Beta’^l^	−3.2813	0.18691	−8.1588	1.5962
‘P3_S1_Beta’^m^	3.1801	0.23433	−2.0664	8.4267
‘P4_S2_Beta’^n^	**7**.**6183**	**0.00198** **	**2**.**802**	**12**.**435**
‘P3_S2_Beta’^o^	−3.9588	0.11565	−8.8935	0.97588

Model specification: Linear mixed-effect model for reaction times during encoding				
Fixed effect coefficients	Estimate (coefficients)	*P* value	Lower (95% CI)	Upper (95% CI)
‘Intercept’	0.64949	0	0.58983	0.70917
‘Group_NF’	−0.03142	0.31852	−0.09324	0.03039
‘Reward’	−0.01174	0.14214	−0.02744	0.00394
‘Day’	**−0**.**04623**	**9.9032e−14** ***	**−0**.**05814**	**−0**.**03433**
‘P4_S1_Alpha’	−0.01059	0.45378	−0.03835	0.01716
‘P3_S1_Alpha’	0.00375	0.79914	−0.02522	0.03273
‘P4_S2_Alpha’	0.01955	0.16865	−0.00830	0.04741
‘P3_S2_Alpha’	−0.01761	0.22544	−0.04613	0.01089
‘P4_S1_Theta’	**−0**.**02738**	**0.04818** *	**−0**.**05455**	**−0**.**00021**
‘P3_S1_Theta’	0.01868	0.17813	−0.00853	0.04590
‘P4_S2_Theta’	0.00802	0.57443	−0.02002	0.03606
‘P3_S2_Theta’	−0.00359	0.7913	−0.03023	0.02304
‘P4_S1_Beta’	−0.01251	0.44115	−0.04441	0.01937
‘P3_S1_Beta’	0.01866	0.2914	−0.01605	0.05338
‘P4_S2_Beta’	−0.01792	0.27006	−0.04981	0.01395
‘P3_S2_Beta’	0.00569	0.72904	−0.02658	0.03798

The results from the full H_1_ based on restricted maximum likelihood (REML) are reported for accuracy and RTs during the encoding period. Fixed effect coefficients and related estimates, *P* values, and lower and upper 95% confidence intervals (CIs) are reported. Only the first 1000 ms of the encoding period was considered for this analysis and split into equal segments of 500 ms (S1 from 0 to 500 ms, S2 from 500 to 1000 ms relative to the onset of the sample object). P3: left parietal electrode. P4: right parietal electrode.

a Fixed effect coefficient ‘group’ with 2 levels: NF group and CO group.

b Fixed effect coefficient ‘reward’ with 2 levels: Reward and No-Reward conditions.

c Fixed effect coefficient ‘day’ with 5 levels: 1, 2, 3, 4, and 5.

d Fixed effect coefficient for alpha frequency at channel P4 during the encoding period from 0 to 500 ms.

e Fixed effect coefficient for alpha frequency at channel P3 during the encoding period from 0 to 500 ms.

f Fixed effect coefficient for alpha frequency at channel P4 during the encoding period from 500 to 1000 ms.

g Fixed effect coefficient for alpha frequency at channel P3 during the encoding period from 500 to 1000 ms.

h Fixed effect coefficient for theta frequency at channel P4 during the encoding period from 0 to 500 ms.

I Fixed effect coefficient for theta frequency at channel P3 during the encoding period from 0 to 500 ms.

j Fixed effect coefficient for theta frequency at channel P4 during the encoding period from 500 to 1000 ms.

k Fixed effect coefficient for theta frequency at channel P3 during the encoding period from 500 to 1000 ms.

l Fixed effect coefficient for beta frequency at channel P4 during the encoding period from 0 to 500 ms.

m Fixed effect coefficient for beta frequency at channel P3 during the encoding period from 0 to 500 ms.

n Fixed effect coefficient for beta frequency at channel P4 during the encoding period from 500 to 1000 ms.

o Fixed effect coefficient for beta frequency at channel P3 during the encoding period from 500 to 1000 ms.

* Significant effect of right parietal theta power at S1 with *P* < 0.05.

** Significant effect of reward anticipation and right parietal beta power at S2 with *P* < 0.01.

*** Significant effect of training with *P* < 0.001.

**Table 2:** Model specification and fixed effect coefficients with related statistics

Model specification: Linear mixed-effect model for accuracy during maintenance				
Fixed effect coefficients	Estimate (coefficients)	*P* value	Lower (95% CI)	Upper (95% CI)
‘Intercept’	55.584	0	51.064	60.105
‘Group_NF’	−0.11841	0.95248	−4.0193	3.7824
‘Reward’	**3**.**2822**	**0.01065** **	**0**.**76602**	**5**.**7984**
‘Day’	**3**.**5565**	**4.174e−12** ***	**2**.**5699**	**4**.**5431**
‘P4_Alpha’^a^	−7.0532	0.15263	−16.726	2.6194
‘P3_Alpha’^b^	2.8494	0.56817	−6.9503	12.649
‘P4_Theta’^c^	3.2075	0.45836	−5.2823	11.697
‘P3_Theta’^d^	−4.5575	0.22225	−11.883	2.7683
‘P4_Beta’^e^	8.0678	0.20412	−4.3961	20.535
‘P3_Beta’^f^	−0.57879	0.93406	−14.313	13.155

Model specification: Linear mixed-effect model for reaction times during maintenance				
Fixed effect coefficients	Estimate (coefficients)	*P* value	Lower (95% CI)	Upper (95% CI)
‘Intercept’	0.64791	0	0.58883	0.70699
‘Group_NF’	−0.03491	0.26923	−0.09691	0.02709
‘Reward’	−0.01115	0.1499	−0.02635	0.00404
‘Day’	**−0**.**04586**	**1.4744e−13** ***	**−0**.**05776**	**−0**.**03396**
‘P4_Alpha’	0.01460	0.67175	−0.05306	0.08227
‘P3_Alpha’	0.02065	0.54877	−0.04695	0.08826
‘P4_Theta’	−0.04588	0.10875	−0.10199	0.01022
‘P3_Theta’	−0.02420	0.3202	−0.07198	0.02357
‘P4_Beta’	**−0**.**08996**	**0.03132** ^*^	**−0**.**17183**	**−0**.**00809**
‘P3_Beta’	**0**.**09322**	**0.04578** ^*^	**0**.**00175**	**0**.**18469**

The results from the full H_1_ based on restricted maximum likelihood (REML) are reported for accuracy and RTs during the maintenance period (20 s). Fixed effect coefficients and related coefficient estimates, *P* values, and lower and upper 95% confidence intervals (CIs) are reported. The entire maintenance period (20 s) was considered for this analysis. P3: left parietal electrode. P4: right parietal electrode.

a Fixed effect coefficient for alpha frequency at channel P4 during the entire maintenance period.

b Fixed effect coefficient for alpha frequency at channel P3 during the entire maintenance period.

c Fixed effect coefficient for theta frequency at channel P4 during the entire maintenance period.

d Fixed effect coefficient for theta frequency at channel P3 during the entire maintenance period.

e Fixed effect coefficient for beta frequency at channel P4 during the entire maintenance period.

f Fixed effect coefficient for beta frequency at channel P3 during the entire maintenance period.

* Significant effect of parietal beta power at channel P3 and P4 with *P* < 0.05.

** Significant effect of reward anticipation with *P* < 0.01.

*** Significant effect of training with *P* < 0.001.

#### Accuracy

During encoding, no significant NF-training effect on improved performance was found (Supplementary Material 4.1). However, we found that reward anticipation led to improved accuracy (Supplementary Fig. 1A). Moreover, a significant effect of right parietal beta power at S2 indicated that an increase in relative power was related to improved accuracy across 5 days (Supplementary Fig. 1.B). This was not the case for beta oscillations during the maintenance phase.

*Post hoc* analyses (Supplementary Table 1) showed a significant interaction between reward anticipation and right parietal beta at S2, indicating that lower beta values were positively related to accuracy when a reward was expected (Supplementary Fig. 2).

The ML model fit indices revealed that H_1_ did differ significantly from H_0_ during encoding (*χ*^2^ (14) = 26.68, *P* = 0.0211) but not maintenance (*χ*2 (8) = 12.101, *P* = 0.1467). The Akaike correction criteria (AIC) of H_1_ was lower (4984.3) during encoding but higher during maintenance (4986.9) than H_0_ (4983). The adjusted *R*^2^ of H_1_ during encoding (0.1936) and maintenance (0.1840) was higher than that of H_0_ (0.1740). Hence, H_1_ demonstrated a better model fit during encoding. Furthermore, the interaction model H_1_ did differ significantly from H_0_ during encoding (*χ*2 (6) = 18.86, *P* = 0.0044). The AIC of H_1_ was lower (4976.1) during encoding than H_0_. The adjusted *R*^2^ of H_1_ (0.1972) was higher than that of H_0_. Hence, the interaction model H_1_ demonstrated a better model fit.

#### Reaction times

During encoding, no significant NF-training effects on mean RTs were found (Supplementary Fig. 3A). Moreover, a significant effect of right parietal theta power at S1 indicated that an increase in relative power at encoding was related to faster RTs across 5 days (Supplementary Fig. 3B).

For the maintenance phase, we found a significant effect of bilateral parietal beta power. The RTs were found to be negatively related to the right parietal electrode and positively related to the left parietal electrode. Thus, an increase in relative power at P4 across 5 days was associated with faster RTs, and the opposite was the case for P3 (Supplementary Fig. 4).

*Post hoc* analyses (Supplementary Table 1) showed that the interaction between group and right parietal theta at S1 was significant, indicating that an increase in theta power in the NF group was related to faster RTs (Supplementary Fig. 5). For the maintenance phase, *post hoc* analyses showed no significant interactions (Supplementary Table 2).

The ML model fit indices revealed that H_1_ did not differ significantly from H_0_ during encoding (*χ*^2^ (14) = 15.888, *P* = 0.3202) but did differ significantly in maintenance (*χ*^2^ (8) = 18.241, *P* = 0.0194). The AIC of H_1_ was higher (−833.27) during encoding but lower in maintenance (−847.63) than H_0_ (−845.38). The adjusted *R*^2^ of H_1_ during encoding (0.7304) and maintenance (0.7359) was higher than that of H_0_ (0.7283). Hence, H_1_ demonstrated a better model fit during maintenance. Furthermore, the interaction model H_1_ did differ significantly from H_0_ during encoding (*χ*^2^ (5) = 15.887, *P* = 0.0071) but not in maintenance (*χ*^2^ (9) = 15.316, *P* = 0.0826). The AIC of H_1_ was lower (−851.27) during encoding but higher in maintenance (−842.7) than that of H_0_. The adjusted *R*^2^ of H_1_ during encoding (0.7339) and maintenance (0.7347) was higher than that of H_0_. Hence, the interaction model H_1_ demonstrated a better model fit during encoding.

## Discussion

We conducted two studies to investigate the hypothesis that NF-training of alpha suppression would improve WM performance, particularly when anticipating a reward, and that NF-training would also facilitate the performance of unrelated cognitive tasks by transfer effects. Moreover, we also tested whether different mental strategies during maintenance would differentially affect alpha oscillations and consequently WM. Hence, in Study I, participants were engaged in MC during maintenance, whereas in Study II, they were engaged in MI of the to-be-maintained sample. We also investigated the effect of cognitive training on theta and beta oscillations across both studies. Lastly, we performed an LMM analysis to investigate any relationships between WM performance and oscillations at encoding and maintenance across training days.

The results from Study I showed a significant training and reward-anticipation effect on WM with improved accuracy and RTs. However, no differences in performance were found between groups; thus, WM improvements were unrelated to NF-training effects. Although we found a decrease in alpha power over days, it was not significant and not consistent in both groups. In addition, no differences in alpha power were related to reward anticipation. Thus, neither NF-training nor reward-anticipation effects on alpha suppression were found. We explained the observed alpha power decrease in CO due to placebo effects;^51^ indeed, this group was treated as an active control receiving the same experimental instructions, potentially influencing our results, as suggested by previous findings.^52^ Hence, our results might contrast with previous studies^10^ that found a reward-anticipation effect on alpha suppression and improved WM performance in the DMST.^2^ Moreover, we found an NF-training and reward-anticipation effect on enhancing theta suppression over days. Indeed, previous studies reported that theta activity was related to the reward system of the brain, actively engaged during learning,^53^ and strongly modulated by reward anticipation, consequently facilitating learning and memory formation.^54,55^ However, although theta activity was modulated by NF-training of alpha suppression, the observed decrease in power during maintenance contrasted with previous findings.^56,57^ Furthermore, we found low beta suppression, which was not related to either NF-training or reward anticipation effects. Which cognitive aspect of our WM task was related to beta suppression remains unclear. A persistent decrease in parietal alpha and beta oscillations during encoding and maintenance was previously associated with attention processes while performing a WM task.^58^ Other studies related the strong beta suppression to a mechanism of motor-cortical activity and successfully related it to learning and RT gain.^58,59^ In addition, alpha and beta activities were also found to be correlated with memory encoding and retrieval in an old/new recognition paradigm.^16^ A partly different outcome was observed in Study II. Despite a significant training and reward-anticipation effect on WM, no differences in performance between groups were found. Neither significant reward-anticipation nor NF-training effects on alpha-suppression, theta, or beta oscillations were observed. Thus, the MC^60-62^ strategy might have been more efficient than MI in modulating oscillations during maintenance for reasons we do not yet understand. It is known that 10–50% of individuals fail to control NF signals across sessions.^63-65^ Therefore, a better understanding is needed of which mental strategy is more effective to enhance NF-learning.^65^ In addition, even targeting either the lower or upper alpha frequencies did not show the expected effect on NF and cognitive performances as reported by previous studies.^5,12,16-18,66^ Moreover, changing the range of trained frequencies across days might have compromised the NF-training preventing participants to develop a strategy (see Supplementary Material 6). Furthermore, no transfer effects were observed across studies. Overall, despite a lack of effect of NF-training on oscillations, questionnaire results showed that in both groups and studies, participants perceived improvements in memory due to the training, reporting an improved concentration after the experiment.

Lastly, we conducted an exploratory analysis across both studies to investigate any relationships between WM improvements and specific oscillatory changes during encoding and maintenance across days. The LMM analysis revealed that during encoding, the increase in right parietal low-beta after 500 ms of stimulus onset was related to improved mean accuracy, with a significant reward-anticipation effect. An interaction effect was also found, showing how the modulation of beta may influence performance when expecting a reward. In contrast, the increase in right parietal theta at encoding and over days was related to faster mean RTs.^67^ Similar to previous findings,^47,48^ the observed increase in theta rapidly occurred after stimulus onset (i.e. within 500 ms in our study), and this power increase might be related to the obtained improved memory performances. However, based on the model comparison, this result was not strongly supported. Additionally, in a *post hoc* analysis, NF-training was found to modulate theta oscillations while influencing RTs, showing how the power of theta oscillations is related to successful encoding. Additionally, as found in previous studies,^68-70^ no specific oscillations were related to accuracy during maintenance. However, bilateral parietal beta amplitudes were related to mean RTs, where an increase over the right regions was associated with faster RTs, whereas the opposite relationship was observed over the left hemisphere. The literature shows an inconsistent relationship between WM and beta oscillations. Indeed, an increase in power of parietal beta,^45^ or a decrease,^71,72^ was reported related to memory load and cognitive effort, supporting its contribution to maintenance.^73,74^ In addition, increased beta amplitude was also reported to be related to sensory-motor processing and negatively correlated with enhanced RTs^75^ but also positively related to high-reward expectation in a delayed-response task.^76^

## Conclusions

The WM supports information processing, executive functions, and maintenance of information for goal-directed behaviour. Based on the well-known effect of reward anticipation and alpha suppression on WM, we aimed to improve WM performance by combining NF-training of alpha suppression in monetary-rewarded DMSTs while also investigating the overall effect of cognitive training on the neighbouring frequencies, theta and beta. We also inspected possible transfer effects to unrelated cognitive tasks. Finally, we conducted two studies to compare the effect of implementing different mental strategies during maintenance. We found a reward-anticipation effect on WM performance that was unrelated to NF-training and alpha suppression. Irrespective of NF-training and maintenance instructions, WM improvements were related to parallel changes in beta oscillations during encoding and WM accuracy. Therefore, beta oscillations at encoding could be a target for interventions aiming to improve WM performance.

## Supplementary Material

fcad101_Supplementary_Data

## Abbreviations

AIC =: Akaike correction criteria
CO =: control
CT =: cognitive training
DMST =: delayed match-to-sample task
FA =: false alarm rates
FFT =: fast-Fourier transform
H =: hit rates
LMM =: linear mixed-effect model
LSL =: lab streaming layer
M =: mean
MC =: mental calculation
MD =: mean difference
Mdn =: median
MI =: mental imagery
ML =: maximum likelihood
MST =: mnemonic similarity test
NF =: neurofeedback
REML =: restricted maximum likelihood
RTs =: reaction times
SEM =: standard error
SD =: standard deviation
WM =: working memory
