# Supplementary material for: Cognitive training, but not EEG-neurofeedback, improves working memory in healthy volunteers
Source: Brain Commun. 2023 Mar 30;5(2):fcad101. doi: 10.1093/braincomms/fcad101 (PMC10082554; doi:10.1093/braincomms/fcad101)
Supplement: fcad101_Supplementary_Data [file fcad101_supplementary_data.docx]

**SUPPLEMENTARY MATERIAL**

**Contents**

**Pg. 3-4** **Supplementary Material 1 -** **Statistical analysis**

**Pg. 3** **Supplementary Material 1.1** - Sample size

**Pg. 3**  **Supplementary Material 1.2** - Study I and II (Transfer effect tasks)

**Pg. 3-4** **Supplementary Material 1.3** - Combined analysis across studies

**Pg. 4-8** **Supplementary Material 2 - Additional results Study I**

**Pg. 4-5**  **Supplementary Material 2.1** - Additional statistics on DMST

**Pg. 5-6** **Supplementary Material 2.2** - Additional statistics on alpha power (7-13 Hz)

**Pg. 6**-7 **Supplementary Material 2.3** - Additional statistics on theta power (4-7 Hz)

**Pg. 7-8** **Supplementary Material 2.4** - Additional statistics on low-beta power (13-20 Hz)

**Pg. 8** **Supplementary Material 2.5** - Additional statistics on transfer effect tasks

**Pg. 8** **Supplementary Material 2.6** - Statistics from the questionnaire

**Pg. 9-13** **Supplementary Material 3 - Additional results Study II**

**Pg. 9**  **Supplementary Material 3.1** - Additional statistics on DMST

**Pg. 9-10** **Supplementary Material 3.2** - Additional statistics on alpha power (7-13 Hz)

**Pg. 10-11** **Supplementary Material 3.3** - Additional statistics on theta power (4-7 Hz)

**Pg. 11-12** **Supplementary Material 3.4** - Additional statistics on low-beta power (13-20 Hz)

**Pg. 12** **Supplementary Material 3.5** - Additional statistics on transfer effect tasks

**Pg. 12-13** **Supplementary Material 3.6** - Statistics from the questionnaire

**Pg. 13-19** **Supplementary Material 4 - Additional results combined analysis across studies**

**Pg. 13-15** **Supplementary Material 4.1** - Accuracy

**Pg. 14** Supplementary Fig. 1A, Supplementary Fig. 1B

**Pg. 15** Supplementary Fig. 2

**Pg. 15-19** **Supplementary Material 4.2** - RTs

**Pg. 16** Supplementary Fig. 3A, Supplementary Fig. 3B

**Pg. 17** Supplementary Fig. 4A, Supplementary Fig. 4B

**Pg. 18** Supplementary Fig. 5

**Pg. 18-19** Supplementary Table 1

**Pg. 19** Supplementary Table 2

**Pg. 19-20** **Supplementary Material 5 - Questionnaire post-NF-training**

**Pg. 19-20** **Supplementary Material 5.1** - Appendix A

**Pg. 20-21**  **Supplementary Material 6 - Study limitations**

**Pg. 21-22 Supplementary Material 7 - References**

**Supplementary Material 1 - Statistical analysis**

Supplementary Material 1.1 – Sample size

The estimation of the sample size was not based on specific guidelines. Our total sample consisted of 60 participants divided into two studies. Based on power analysis (performed in G*Power^1^) and with a medium effect size of 0.3, the achieved power was 0.777. Similarly, when removing one subject from the analysis (N=59) the power did not change (0.770). In comparison to other studies which involved a smaller sample size^2,3,4^ and considering that our study did involve a training based on 5 days (with a total of 300 EEG recording sessions), the achieved number of our sample can be a proper size to conduct reliable statistical analysis.

Supplementary Material 1.2 – Study I and II (Transfer effect tasks)

To investigate the performance of the MST and Stroop tasks non-parametric statistics were applied. Besides the between-subject analysis, an additional within-subject analysis was performed. Thus, the Wilcoxon Signed Ranks (two-related samples) Test was used. The parameter “T” indicates the sum of ranks, whereas the parameter “z” indicates the Z statistic.

Supplementary Material 1.3 – Combined Analysis Across Studies

Linear mixed effect models (LMM) are a valuable alternative to repeated-measures ANOVAs analysis. Indeed, one advantage when implementing LMM is the possibility to specify random effects (e.g., subjects). Indeed, rather than considering this variance into an error term, LMM can better distribute the variance that is associated with the term that is defined. In addition, differently from the ANOVAs, LMM can also cope with eventual missing data. Hence, several studies have already applied LMM in their analyses.^5,6,7,8^

Models were built based on the restricted maximum likelihood (REML). Alternative hypothesis models (H_1_) included several fixed effect coefficients that were continuous and categorical variables. Continuous variables were “frequency bands” (relative power of alpha, theta, and low-beta) computed for each “channel” location (P4 and P3), during “encoding” (S1 and S2), or “maintenance” (20s), and “day” (day 1 = 1, day 2 = 2, day 3 = 3, day 4 = 4, day 5 = 5). Categorical variables were “reward condition” (2 levels: “reward” (B) compared to “no-reward” (A)), and “group” (2 levels: “NF” (B) compared to “CO” (A)). To investigate WM performance variability across 5 days and among participants, we included a random effect with slope (factor “day”) and intercept vary by participants.

During encoding the H_1_ model included 16 predictors (‘Accuracy (or RTs) ~ 1 + Group + Day + Reward + P4_S1_Alpha + P3_S1_Alpha + P4_S2_Aalpha + P3_S2_Alpha + P4_S1_Theta + P3_S1_Theta + P4_S2_Theta + P3_S2_Theta + P4_S1_Beta + P3_S1_Beta + P4_S2_Beta + P3_S2_Beta + (1 + Day|Subject)’), whereas 10 predictors during maintenance (‘Accuracy (or RTs) ~ 1 + Group + Day + Reward + P4_Alpha + P3_Alpha + P4_Theta + P3_Theta + P4_Beta + P3_Beta + (1 + Day|Subject)’). The statistical significance of H_1_ was compared with a null hypothesis model (H_0_). H_0_ was a nested model of H_1_ which included only 2 predictors (‘Accuracy (or RTs) ~ intercept + Day + (1 + Day|Subject)’) and the same H_1_ random effect. In total, we built 3 models. During encoding and maintenance periods, full models H_1_ were compared with H_0_ while investigating the effect of their predictors on accuracy and RTs (the dependent variables).

Moreover, after observing the significance of the main effects, we performed a post-hoc analysis to investigate possible interaction effects between these significant factors. Thus, 3 models were built to predict mean accuracy and RTs at encoding and maintenance. These models included fixed effects coefficients that were previously significant, interactions between these significant factors, and fixed effects considered relevant for our research. Only accuracy during maintenance was not further investigated because no oscillations were found to be significant in predicting accuracy (e.g., across training days, groups, or reward conditions). During encoding the interaction model H_1_ including 8 predictors (‘Accuracy ~ 1 + Group + Day + Reward + P4_S2_Beta + Group*P4_S2_Beta + Reward*P4_S2_Beta + Day*P4_S2_Beta + (1 + Day|Subject)’) was implemented to assess mean accuracy, whereas the interaction model H_1_ including 7 predictors (‘RTs ~ 1 + Group + Day + Reward + P4_S1_Theta + Group*P4_S1_Theta + Day*P4_S1_Theta + (1 + Day|Subject)’) was implemented to assess mean RTs. During maintenance, the interaction model H_1_ including 11 predictors (‘RTs ~ 1 + Group + Day + Reward + P4_Beta + P3_Beta + Group*P4_Beta + Group*P3_Beta + Day*P4_Beta * Day*P3_Beta + P4_Beta*P3_Beta + (1 + Day|Subject)’) was implemented to assess mean RTs.

The statistical significance of interaction models H_1_ was compared with the same null hypothesis model H_0_. The analysis was performed in MATLAB 2018b.^9^ Graphical representations were made in R software version 1.4.1106.^10,11^ Moreover to compare the mean difference between conditions and groups, a paired sample t-test and independent t-test analyses were applied. The analysis was performed in SPSS software version 26.0.^12^

**Supplementary Material 2 - Additional Results Study I**

All the corrected results from the ANOVA were made with Greenhouse-Geisser. Except where it is stated, the Huynh-Feldt correction was used.

Supplementary Material 2.1 – Additional statistics on DMST

| DMST - Accuracy | | | | | |
| --- | --- | --- | --- | --- | --- |
| Effect | Correction | F | df1 | df2 | p |
| reward*group | Sphericity | 0.047 | 1 | 28 | 0.830 |
| day*reward | Sphericity | 1.761 | 4 | 112 | 0.142 |

| DMST - RTs | | | | | |
| --- | --- | --- | --- | --- | --- |
| Effect | Correction | F | df1 | df2 | p |
| gender | Sphericity | 0.368 | 1 | 27 | 0.549 |
| day*gender | Greenhouse-Geisser | 1.224 | 2.800 | 75.601 | 0.306 |
| reward*gender | Sphericity | 10.019 | 1 | 27 | 0.004 |
| reward*group | Sphericity | 0.047 | 1 | 27 | 0.830 |
| day*reward | Sphericity | 0.875 | 4 | 108 | 0.482 |
| day*reward*gender | Sphericity | 0.736 | 4 | 108 | 0.570 |

Supplementary Material 2.2 – Additional statistics on alpha power (7-13 Hz)

| Alpha (7-13 Hz) | | | | | |
| --- | --- | --- | --- | --- | --- |
| Effect | Correction | F | df1 | df2 | p |
| gender | Sphericity | 2.779 | 1 | 27 | 0.107 |
| channel | Sphericity | 0.615 | 2 | 54 | 0.545 |
| day*gender | Greenhouse-Geisser | 1.278 | 2.543 | 68.672 | 0.288 |
| day*group | Greenhouse-Geisser | 0.443 | 2.543 | 68.672 | 0.691 |
| reward*gender | Sphericity | 0.454 | 1 | 27 | 0.506 |
| reward*group | Sphericity | 0.900 | 1 | 27 | 0.351 |
| channel*gender | Sphericity | 0.895 | 2 | 54 | 0.415 |
| channel*group | Sphericity | 0.359 | 2 | 54 | 0.700 |
| day*reward | Greenhouse-Geisser | 0.936 | 2.617 | 70.668 | 0.418 |
| day*reward*gender | Greenhouse-Geisser | 0.530 | 2.617 | 70.668 | 0.639 |
| day*channel | Greenhouse-Geisser | 1.148 | 4.628 | 124.950 | 0.338 |
| day*channel*gender | Greenhouse-Geisser | 1.211 | 4.628 | 124.950 | 0.309 |
| day*channel*group | Greenhouse-Geisser | 0.978 | 4.628 | 124.950 | 0.430 |
| reward*channel | Sphericity | 0.470 | 2 | 54 | 0.628 |
| reward*channel*gender | Sphericity | 0.447 | 2 | 54 | 0.642 |
| reward*channel*group | Sphericity | 2.001 | 2 | 54 | 0.145 |
| day*reward*channel | Greenhouse-Geisser | 2.156 | 4.569 | 123.365 | .069 |
| day*reward*channel*gender | Greenhouse-Geisser | 1.862 | 4.569 | 123.365 | .0112 |
| day*reward*channel*group | Greenhouse-Geisser | 0.229 | 4.569 | 123.365 | 0.939 |

Supplementary Material 2.3 – Additional statistics on theta power (4-7 Hz)

| Theta (4-7 Hz) | | | | | |
| --- | --- | --- | --- | --- | --- |
| Effect | Correction | F | df1 | df2 | p |
| gender | Sphericity | 2.243 | 1 | 27 | 0.146 |
| channel | Sphericity | 0.783 | 2 | 54 | 0.462 |
| day*gender | Sphericity | 2.196 | 4 | 108 | 0.074 |
| day*group | Sphericity | 0.369 | 4 | 108 | 0.830 |
| reward*gender | Sphericity | 0.241 | 1 | 27 | 0.627 |
| channel*gender | Sphericity | 0.656 | 2 | 54 | 0.523 |
| channel*group | Sphericity | 2.465 | 2 | 54 | 0.094 |
| day*reward | Sphericity | 0.390 | 4 | 108 | 0.816 |
| day*reward*gender | Sphericity | 0.422 | 4 | 108 | 0.793 |
| day*reward*group | Sphericity | 1.311 | 4 | 108 | 0.271 |
| day*channel | Greenhouse-Geisser | 0.730 | 4.535 | 122.446 | 0.590 |
| day*channel*gender | Greenhouse-Geisser | 0.769 | 4.535 | 122.446 | 0.563 |
| day*channel*group | Greenhouse-Geisser | 0.277 | 4.535 | 122.446 | 0.911 |
| reward*channel | Huynh-Feldt | 2.551 | 1.866 | 50.391 | 0.092 |
| reward*channel*gender | Huynh-Feldt | 1.495 | 1.866 | 50.391 | 0.234 |
| reward*channel*group | Huynh-Feldt | 0.227 | 1.866 | 50.391 | 0.782 |
| day*reward*channel | Greenhouse-Geisser | 0.445 | 4.614 | 124.573 | 0.802 |
| day*reward*channel*gender | Greenhouse-Geisser | 0.529 | 4.614 | 124.573 | 0.740 |
| day*reward*channel*group | Greenhouse-Geisser | 0.505 | 4.614 | 124.573 | 0.758 |

Supplementary Material 2.4 – Additional statistics on low-beta power (13-20 Hz)

| Low-beta (13-20 Hz) | | | | | |
| --- | --- | --- | --- | --- | --- |
| Effect | Correction | F | df1 | df2 | p |
| gender | Sphericity | 2.606 | 1 | 27 | 0.118 |
| channel | Huynh-Feldt | 0.836 | 1.843 | 49.757 | 0.431 |
| day*gender | Greenhouse-Geisser | 2.809 | 2.581 | 69.677 | 0.054 |
| day*group | Greenhouse-Geisser | 0.068 | 2.581 | 69.677 | 0.965 |
| reward*gender | Sphericity | 0.908 | 1 | 27 | 0.349 |
| reward*group | Sphericity | 0.524 | 1 | 27 | 0.475 |
| channel*gender | Huynh-Feldt | 0.712 | 1.843 | 49.757 | 0.0485 |
| channel*group | Huynh-Feldt | 2.544 | 1.843 | 49.757 | 0.0093 |
| day*reward | Greenhouse-Geisser | 1.405 | 3.259 | 87.997 | 0.245 |
| day*reward*gender | Greenhouse-Geisser | 1.009 | 3.259 | 87.997 | 0.397 |
| day*channel | Greenhouse-Geisser | 0.344 | 5.303 | 143.169 | 0.894 |
| day*channel*gender | Greenhouse-Geisser | 0.484 | 1.316 | 35.545 | 0.798 |
| day*channel*group | Greenhouse-Geisser | 1.662 | 1.316 | 35.545 | 0.143 |
| reward*channel | Greenhouse-Geisser | 0.021 | 1.316 | 35.545 | 0.934 |
| reward*channel*gender | Greenhouse-Geisser | 0.073 | 1.316 | 35.545 | 0.854 |
| reward*channel*group | Greenhouse-Geisser | 0.392 | 1.316 | 35.545 | 0.592 |
| day*reward*channel | Greenhouse-Geisser | 1.056 | 4.386 | 118.417 | 0.384 |
| day*reward*channel*gender | Greenhouse-Geisser | 0.947 | 4.386 | 118.417 | 0.445 |
| day*reward*channel*group | Greenhouse-Geisser | 0.927 | 4.386 | 118.417 | 0.457 |

Supplementary Material 2.5 - Additional statistics on transfer effect tasks

When investigating transfer effects within-group no significant improvements were found in the NF group. Indeed, MST performance before the training was higher (Mdn = 70.6067) than after the training (Mdn = 68.4460), but the difference was not significant (T = 63.00, z = -.659, p = .510). Moreover, Stroop performance before the training was lower (Mdn = 96.5007) than after the training (Mdn = 96.8093), but the difference was not significant (T = 49.50, z = -.825, p = .410).

When investigating transfer effects within-group no significant improvements were found in the CO group. Indeed, MST performance before the training was lower (Mdn = 65.1933) than after the training (Mdn = 65.3753), but the difference was not significant (T = 61.00, z = -.057, p = .955). Moreover, Stroop performance before the training was lower (Mdn = 93.4047) than after the training (Mdn = 94.7613), but the difference was not significant (T = 73.50, z = -1.319, p = .187).

Supplementary Material 2.6 – Statistics from the questionnaire post-NF-training

Questionnaire results showed that participants judged their memory on day 1 as “slightly not good” (NF group: 33.3%, CO group: 40%). However, they evaluated a “good” memory on day 5 (NF group: 73.3%, CO group: 53.3%), and both groups reported that it was “very important” to show and to reach a good performance (NF group: 66.7%, CO group: 66.7%). Moreover, both groups reported finding memory differences before and after the training (NF and CO group: 100%), estimating changes in “concentration” (NF group: 53.3%, CO group: 53.3%).

Additional results from the questionnaire reported that in both groups participants felt overall “good” (NF group: 46.7%) or “very good” (CO group: 46.7%) and they find the experiment “absolutely not difficult” (NF group: 46.7%,) or “not difficult” (CO group: 40%) to perform. Similarly, both groups did find the instructions “not difficult” (NF group: 53.3 %,) or “absolutely not difficult” (CO group: 66.7%) to understand. Both groups did find it “not difficult” to remember the images presented during the DMST (NF group: 46.7%, CO group: 40%). Both groups applied a neurofeedback strategy (“yes”) to move the while ball in the suggested direction by performing the MC. Furthermore, no suggestions were given to improve the experiment.

**Supplementary Material 3 - Additional Results Study II**

All the corrected results from the ANOVA were made with Greenhouse-Geisser. Except where it is stated, the Huynh-Feldt correction was used.

Supplementary Material 3.1 – Additional statistics on DMST

| DMST - Accuracy | | | | | |
| --- | --- | --- | --- | --- | --- |
| Effect | Correction | F | df1 | df2 | p |
| reward*group | Sphericity | 1.150 | 1 | 28 | 0.293 |
| day*reward | Sphericity | 0.499 | 4 | 112 | 0.736 |

| DMST - RTs | | | | | |
| --- | --- | --- | --- | --- | --- |
| Effect | Correction | F | df1 | df2 | p |
| gender | Sphericity | 0.193 | 1 | 27 | 0.664 |
| day*gender | Greenhouse-Geisser | 0.485 | 2.009 | 54.241 | 0.619 |
| reward*gender | Sphericity | 1.394 | 1 | 27 | 0.248 |
| reward*group | Sphericity | 2.359 | 1 | 27 | 0.136 |
| day*reward | Greenhouse-Geisser | 0.715 | 2.831 | 76.445 | 0.539 |
| day*reward*gender | Greenhouse-Geisser | 0.432 | 2.831 | 76.445 | 0.720 |

Supplementary Material 3.2 – Additional statistics on alpha power (7-13 Hz)

| Alpha (7-13 Hz) | | | | | |
| --- | --- | --- | --- | --- | --- |
| Effect | Correction | F | df1 | df2 | p |
| gender | Sphericity | 0.018 | 1 | 26 | 0.896 |
| channel | Sphericity | 0.561 | 2 | 52 | 0.574 |
| day*gender | Greenhouse-Geisser | 1.592 | 2.908 | 75.602 | 0.200 |
| day*group | Greenhouse-Geisser | 1.839 | 2.908 | 75.602 | 0.149 |
| reward*gender | Sphericity | 0.680 | 1 | 26 | 0.417 |
| reward*group | Sphericity | 1.552 | 1 | 26 | 0.224 |
| channel*gender | Sphericity | 0.862 | 2 | 52 | 0.428 |
| channel*group | Sphericity | 0.118 | 2 | 52 | 0.889 |
| day*reward | Greenhouse-Geisser | 0.958 | 3.148 | 81.860 | 0.420 |
| day*reward*gender | Greenhouse-Geisser | 1.205 | 3.148 | 81.860 | 0.314 |
| day*channel | Greenhouse-Geisser | 0.610 | 2.804 | 72.902 | 0.600 |
| day*channel*gender | Greenhouse-Geisser | 0.552 | 2.804 | 72.902 | 0.637 |
| day*channel*group | Greenhouse-Geisser | 1.276 | 2.804 | 72.902 | 0.289 |
| reward*channel | Huynh-Feldt | 0.271 | 1.769 | 45.984 | 0.737 |
| reward*channel*gender | Huynh-Feldt | 0.181 | 1.769 | 45.984 | 0.809 |
| reward*channel*group | Huynh-Feldt | 3.078 | 1.769 | 45.984 | 0.062 |
| day*reward*channel | Greenhouse-Geisser | 1.695 | 3.123 | 81.188 | 0.173 |
| day*reward*channel*gender | Greenhouse-Geisser | 1.347 | 3.123 | 81.188 | 0.264 |
| day*reward*channel*group | Greenhouse-Geisser | 0.659 | 3.123 | 81.188 | 0.585 |

Supplementary Material 3.3 – Additional statistics on theta power (4-7 Hz)

| Theta (4-7 Hz) | | | | | |
| --- | --- | --- | --- | --- | --- |
| Effect | Correction | F | df1 | df2 | p |
| gender | Sphericity | 1.849 | 1 | 26 | 0.186 |
| channel | Greenhouse-Geisser | 0.224 | 1.174 | 30.533 | 0.679 |
| day*gender | Sphericity | 0.494 | 4 | 104 | 0.740 |
| day*group | Sphericity | 2.055 | 4 | 104 | 0.092 |
| reward*gender | Sphericity | 0.091 | 1 | 26 | 0.766 |
| reward*group | Sphericity | 0.513 | 1 | 26 | 0.480 |
| channel*gender | Greenhouse-Geisser | 0.316 | 1.174 | 30.533 | 0.614 |
| channel*group | Greenhouse-Geisser | 0.674 | 1.174 | 30.533 | 0.441 |
| day*reward | Sphericity | 1.284 | 4 | 104 | 0.281 |
| day*reward*gender | Sphericity | 1.070 | 4 | 104 | 0.375 |
| day*channel | Greenhouse-Geisser | 1.341 | 1.317 | 34.235 | 0.265 |
| day*channel*gender | Greenhouse-Geisser | 0.748 | 1.317 | 34.235 | 0.428 |
| day*channel*group | Greenhouse-Geisser | 1.930 | 1.317 | 34.235 | 0.171 |
| reward*channel | Greenhouse-Geisser | 0.921 | 1.192 | 30.990 | 0.362 |
| reward*channel*gender | Greenhouse-Geisser | 0.658 | 1.192 | 30.990 | 0.449 |
| reward*channel*group | Greenhouse-Geisser | 1.682 | 1.192 | 30.990 | 0.206 |
| day*reward*channel | Greenhouse-Geisser | 1.777 | 1.378 | 35.841 | 0.191 |
| day*reward*channel*gender | Greenhouse-Geisser | 1.051 | 1.378 | 35.841 | 0.336 |
| day*reward*channel*group | Greenhouse-Geisser | 1.219 | 1.378 | 35.841 | 0.293 |

Supplementary Material 3.4 – Additional statistics on low-beta power (13-20 Hz)

| Low-Beta (13-20 Hz) | | | | | |
| --- | --- | --- | --- | --- | --- |
| Effect | Correction | F | df1 | df2 | p |
| gender | Sphericity | 0.301 | 1 | 26 | 0.588 |
| channel | Sphericity | 0.041 | 2 | 52 | 0.960 |
| day*gender | Greenhouse-Geisser | 0.528 | 2.903 | 75.481 | 0.658 |
| day*group | Greenhouse-Geisser | 1.904 | 2.903 | 75.481 | 0.138 |
| reward*gender | Sphericity | 3.990 | 1 | 26 | 0.056 |
| reward*group | Sphericity | 0.014 | 1 | 26 | 0.905 |
| channel*gender | Sphericity | 0.245 | 2 | 52 | 0.784 |
| channel*group | Sphericity | 0.544 | 2 | 52 | 0.583 |
| day*reward | Greenhouse-Geisser | 0.498 | 2.720 | 70.731 | 0.667 |
| day*reward*gender | Greenhouse-Geisser | 0.447 | 2.720 | 70.731 | 0.701 |
| day*channel | Greenhouse-Geisser | 0.936 | 3.378 | 87.839 | 0.435 |
| day*channel*gender | Greenhouse-Geisser | 0.764 | 3.378 | 87.839 | 0.532 |
| day*channel*group | Greenhouse-Geisser | 0.748 | 3.378 | 87.839 | 0.541 |
| reward*channel | Sphericity | 0.143 | 2 | 52 | 0.867 |
| reward*channel*gender | Sphericity | 0.731 | 2 | 52 | 0.486 |
| reward*channel*group | Sphericity | 1.309 | 2 | 52 | 0.279 |
| day*reward*channel | Greenhouse-Geisser | 0.868 | 3.565 | 92.684 | 0.476 |
| day*reward*channel*gender | Greenhouse-Geisser | 0.537 | 3.565 | 92.684 | 0.689 |
| day*reward*channel*group | Greenhouse-Geisser | 0.717 | 3.565 | 92.684 | 0.567 |

Supplementary Material 3.5 – Additional statistics on transfer effect tasks

When investigating transfer effects within-group no significant improvements were found in the NF group. Indeed, the MST performance before the training was lower (Mdn = 62.2960) than after the training (Mdn = 62.7307), but the difference was not significant (T = 44.00, z = -.909, p = .363). Moreover, Stroop performance before the training was lower (Mdn = 94.1187) than after the training (Mdn = 94.9040), but the difference was not significant (T = 56.00, z = -1.338, p = .181).

When investigating transfer effects within-group no significant improvements were found in the CO group regarding the MST. Indeed, the MST performance before the training was higher (Mdn = 63.6507) than after the training (Mdn = 60.7473), but the difference was not significant (T = 44.00, z = -.909, p = .363). However, Stroop performance improved. Indeed, the performance before the training was lower (Mdn = 92.9753) than after the training (Mdn = 95.0460), and this difference was significant (T = 77.50, z = -2.238, p = .025).

Supplementary Material 3.6 – Statistics from the questionnaire post-NF-training

Questionnaire results showed that participants judged their memory on day 1 as “slightly not good” (NF group: 40%, CO group: 50%), but differently, they evaluated a “good” memory on day 5 (NF group: 53.3%, CO group: 43.8%). Both groups reported that it was “important” (NF group: 60%) or “very important” (CO group: 43.8%) to show and to achieve good performance. Both groups reported finding memory differences before and after the training (NF and CO group: 100%), estimating changes in “concentration” (NF group: 40%, CO group: 56.3%).

Additional results from the questionnaire reported that in both groups participants felt overall “good” (NF group: 46.7%) or “very good” (CO group: 68.8%) and they find the experiment “slightly not difficult” (NF group: 40%) or “not difficult” (CO group: 56.3%) to perform. Similarly, both groups did find the instructions “absolutely not difficult” (NF group: 46.7 %) or “not difficult” (CO group: 37.5%) to understand. Both groups did find it “not difficult” to remember the images presented during the DMST (NF group: 40%, CO group: 31.3%). Both groups applied a neurofeedback strategy (“yes”) to move the white ball in the suggested direction by performing a MI. No suggestions were given to improve the experiment.

**Supplementary Material 4 - Additional Results Combined Analysis Across Studies**

Supplementary Material 4.1 – Accuracy

During encoding, while investigating the relationship between improved mean accuracy across 5 days and groups, the negative coefficient (𝛽 = -0.93638) indicates that although the increase in accuracy was higher in the CO group (M = 68.1400, SD = 17.03148) than the NF group (M = 67.4615, SD = 17.90666), the difference was not significant (MD = -.67857, SEM = 1.43965, t_588_ = -.471, p = .638, CI[-3.50605, 2.14892]). Thus, we did not find a significant NF-training effect on improved mean accuracy. We also found that improved mean accuracy was positively related with reward-anticipation (𝛽 = 3.3522, p = .01). Indeed, mean accuracy in reward trials was higher (M = 69.4523, SD = 16.49847) than no-reward trials (M = 66.1377, SD = 18.26908), and the difference was significant (MD = 3.31468, SEM = 1.43321, t_588_ = 2.313, p = .021, CI[.49984, 6.12952]) [Supplementary Fig. 1.A].

Moreover, we found that improved mean accuracy was related to the relative mean power of right parietal beta at S2 (from 500ms to 1000ms after stimulus onset). The positive estimate (𝛽 = 7.6183) indicates that an increase of right parietal beta power among all participants was related to increased mean accuracy across 5 days and that the increase was significant (p = 0.001) [Supplementary Fig. 1.B]. Thus, we compared the relative mean power of beta in the S2 with S1 time windows. Indeed, beta power at S2 (M = -.0017, SD = .46163) was higher than at S1 (M = -.0084, SD = .44710) across 5 days, although the difference in power was not significant (MD = -.00666, SEM = .01619, t_589_ = -.411, p = .681, CI[-.03845,.02514]).

From the post-hoc analysis [Supplementary Table 1], the interaction between reward-anticipation and right parietal beta at S2 was significant, indicating that lower values of beta power would positively influence accuracy, but only when a reward is expected. Thus, a reward-anticipation effect on accuracy and beta oscillations was found [Supplementary Fig. 2]. The mean beta power across 5 days was lower in reward (M = -.0216, SD = .47124), and higher in no-reward (M = .0182, SD = .45173) trials, although the difference was not significant (MD = -.03982, SEM = 0.3801, t_588_ = -1.048, p = .295, CI[-.11447, .03483]).


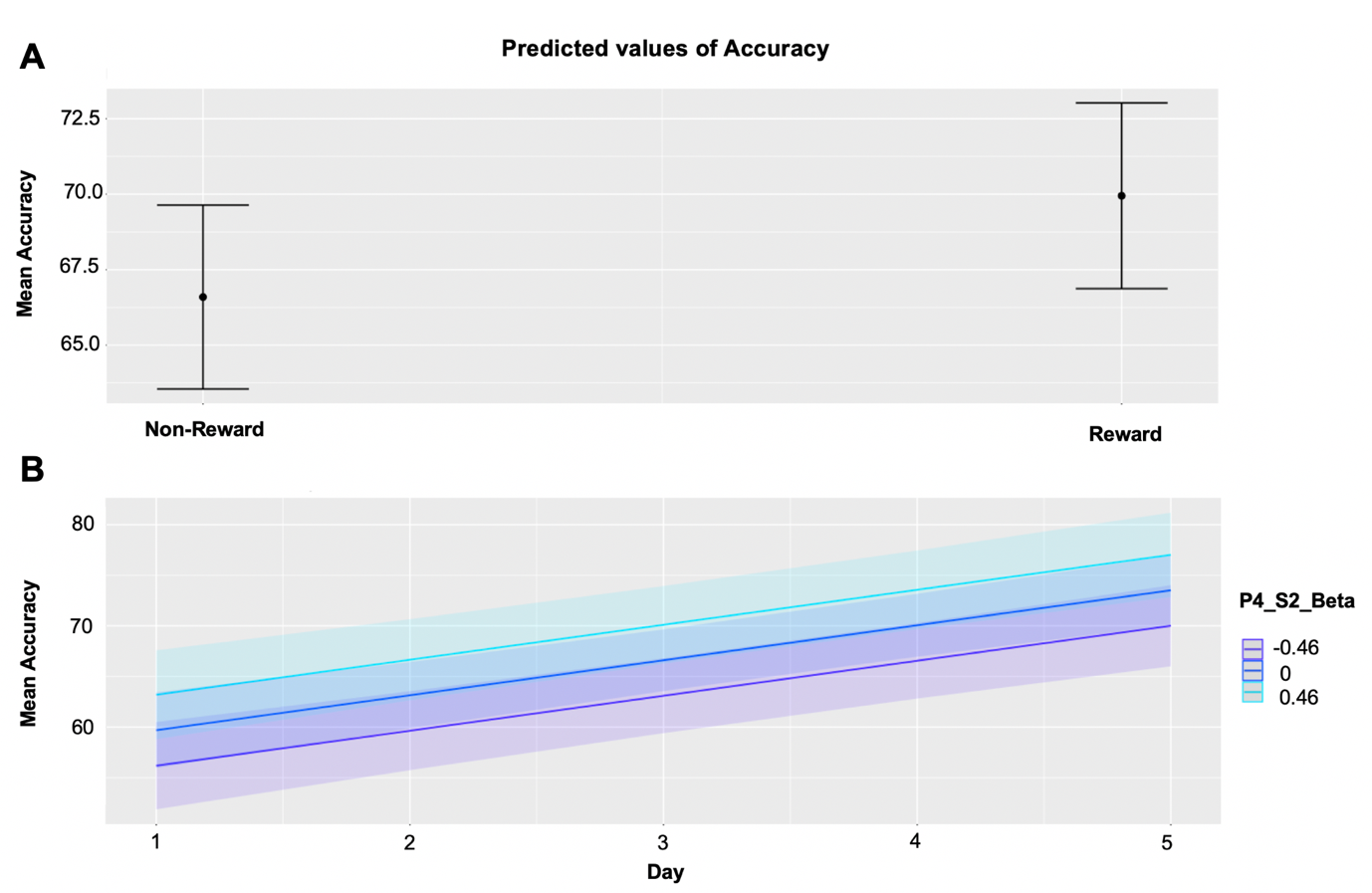


**Supplementary Fig. 1 Predicted values of Accuracy during encoding.** Both figures show results from the full H_1_ model based on restricted maximum likelihood (REML) identifying accuracy as the target variable during encoding. **A** Predicted values of accuracy are represented for the reward (right) and for the non-reward (left) condition during encoding. Reward conditions are represented on the x-axis and mean accuracy on the y-axis. Standard error of the mean is denoted by the error bars. Results showed a significant main effect of reward indicating that reward anticipation led to improved accuracy (𝛽 = 3.3522, p = .01, CI [0.79302, 5.9115]). **B** Predicted values of accuracy are represented in relation to the relative power of right parietal beta at time window S2, across 5 days. The relative power of beta across 5 days is represented on the x-axis and mean accuracy on the y-axis. Standard error of the mean defines the confidence intervals. The light blue line indicates higher values of beta power. The blue line indicates values around zero of beta power. The purple line indicates lower values of beta power. Results showed a significant effect of right parietal beta power at S2 which indicated that an increase in relative power was related to improved accuracy across 5 days (𝛽 = 7.6183, p = 0.001, CI[2.802, 12.435]).


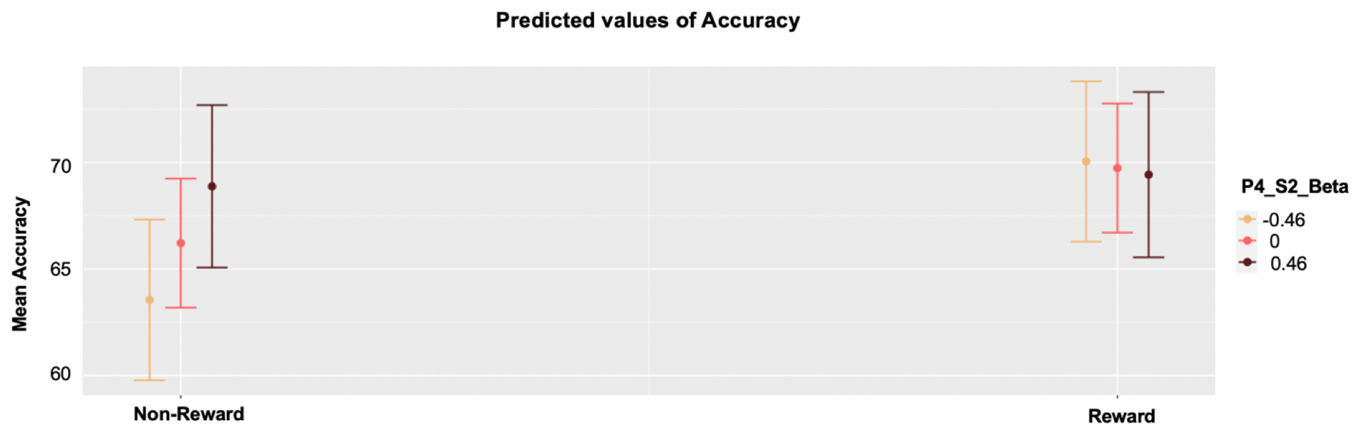


**Supplementary Fig. 2 Predicted values of Accuracy during encoding with interaction effects.** The figure shows results from the interaction H_1_ model based on restricted maximum likelihood (REML) identifying accuracy as the target variable during encoding. Predicted values of accuracy are represented for the reward (right) and for the non-reward (left) condition and in relation to the relative power of right parietal beta at time window S2 during encoding. Reward conditions are represented on the x-axis in relation to the relative power of beta and mean accuracy on the y-axis. Standard error of the mean is denoted by the error bars. The brown line indicates higher values of beta power. The pink line indicates values around zero of beta power. The yellow line indicates lower values of beta power. Results showed a significant interaction between reward anticipation and right parietal beta at S2, indicating that lower beta values were positively related to accuracy when a reward was expected (𝛽 = -6.4747, p = 0.023, CI[-12.066, -0.88352]).

Supplementary Material 4.2 – RTs

During encoding, when investigating the relationship between improved mean RTs across the training and groups, the negative coefficient (𝛽 = -0.03142) indicates that although the decrease in mean RTs was higher in the NF group (M = .4722, SD = .17491) than the CO group (M = .5058, SD = .17063), the difference was not significant in the model (p = .318). However, from a (independent sample) t-test analysis the difference was significant (MD = -.03363, SEM = .01423, t_588_ = -2.363, p = .018, CI[-.06158, -.00568]) [Supplementary Fig. 3A]. Thus, we did not find a significant NF-training effect on improved mean RTs in the full H_1_ model, but potentially the NF-training might have an effect on improving RTs performances. Moreover, faster RTs were found related to the relative power of the right parietal theta at S1 (within 500ms after stimulus onset). The negative estimate (𝛽 = -0.02738) indicates that an increase of relative theta power at the right parietal region, and across all participants, was related to faster RTs and that the increase was significant (p = 0.048) [Supplementary Fig. 3B]. Thus, we compared the relative mean power of theta oscillations in the S1 with S2 time windows. The mean theta power at S1 (M = -.0120, SD = .51898) was higher than at S2 (M = -.0176, SD = .50695) across 5 days, although the difference was not significant (MD = .00566, SEM = .01804, t_589_ = .314, p = 754, CI[-.02978, .04110]).


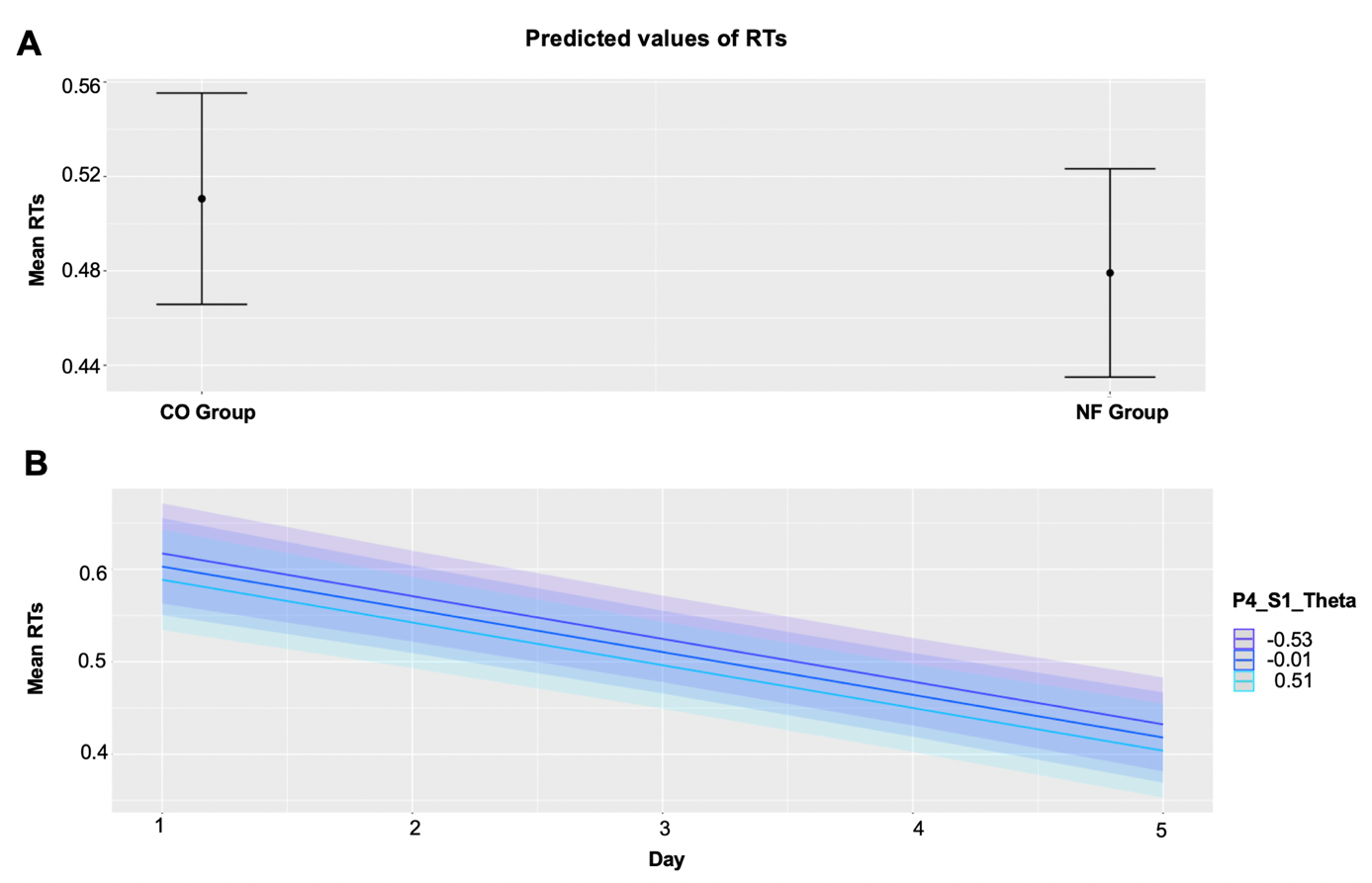


**Supplementary Fig. 3 Predicted values of RTs during encoding.** Both figures show results from the full H_1_ model based on restricted maximum likelihood (REML) identifying RTs as the target variable during encoding**. A** Predicted values of RTs are represented for the NF group (right) and for the CO group (left) during encoding. Groups are represented on the x-axis and mean RTs on the y-axis. Standard error of the mean is denoted by the error bars. Results showed no significant NF-training effects on mean RTs (𝛽 = -0.03142, p = .318, CI[-0.09324, 0.03039]). **B** Predicted values of RTs are represented in relation to the relative power of right parietal theta at time window S1, across 5 days. The relative power of theta across 5 days is represented on the x-axis and mean RTs on the y-axis. Standard error of the mean defines the confidence intervals. The light blue line indicates higher values of theta power. The blue line indicates values around zero of theta power. The purple line indicates lower values of theta power. Results showed a significant effect of right parietal theta power at S1 indicating that an increase in relative power at encoding was related to faster RTs across 5 days (𝛽 = -0.02738, p = 0.048, CI[-0.05455, -0.00021]).

During maintenance, no differences in RT performance were found between groups (𝛽 = -0.03491, p = .269). Moreover, mean RTs were found negatively related with the relative power of parietal beta at P4 (𝛽 = -0.08996, p = .0313) and positively related with relative power of parietal beta at P3 (𝛽 = 0.09322, p = .045). Thus, an increase of relative power at P4 across 5 days would be associated with consequent faster RTs, whereas the opposite relation would occur over left parietal beta at P3 where an increase in relative beta power would affect RTs while slowing the performance [Supplementary Fig. 4.A, 4.B]. Overall the relative power of beta at P4 was slightly lower (M = .0014, SD = .24117) than at P3 (M = .0086, SD = .22571), although the difference was not significant (MD = -.00712, SEM = .00523, t_589_ = -1.360, p = .174, CI[-.01740, .00316]).


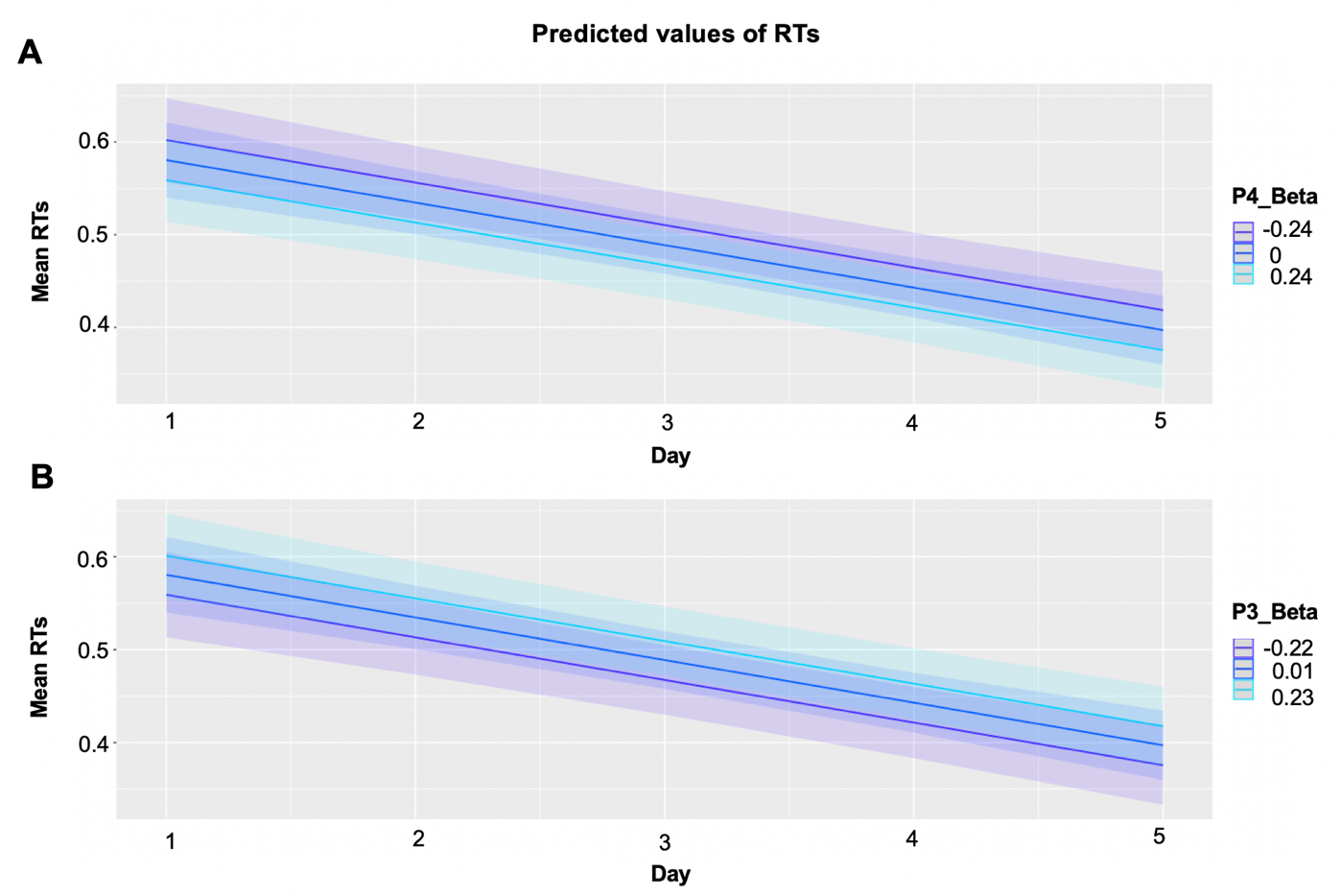


**Supplementary Fig. 4 Predicted values of RTs during maintenance.** Both figures show results from the full H_1_ model based on restricted maximum likelihood (REML) identifying RTs as the target variable during maintenance**. A** Predicted values of RTs are represented in relation to the relative power of right parietal beta at P4 across 5 days. The relative power of beta across 5 days is represented on the x-axis and mean RTs on the y-axis. Standard error of the mean defines the confidence intervals. The light blue line indicates higher values of beta power at P4. The blue line indicates values around zero of beta power at P4. The purple line indicates lower values of beta power at P4. Results showed that RTs were negatively related to the right parietal electrode and thus an increase in relative power at P4 across 5 days was associated with faster RTs (𝛽 = -0.08996, p = .0313, CI[-0.17183, -0.00809]). **B** Predicted values of RTs are represented in relation to the relative power of left parietal beta at P3 across 5 days. The relative power of beta across 5 days is represented on the x-axis and mean RTs on the y-axis. Standard error of the mean defines the confidence intervals. The light blue line indicates higher values of beta power at P3. The blue line indicates values around zero of beta power at P3. The purple line indicates lower values of beta power at P3. Results showed that RTs were positively related to the left parietal electrode and thus an increase in relative power at P3 across 5 days was associated with slower RTs (𝛽 = 0.09322, p = .045, CI[0.00175, 0.18469]).

From the post-hoc analysis [Supplementary Table 1], the interaction between group and right parietal theta at S1 was significant. This indicates that an increase in theta power observed in the NF-group would affect the mean RTs. Thus, an NF-training effect on RTs and theta oscillations was found [Supplementary Fig. 5]. Overall, the mean relative power of theta across 5 days was higher in NF (M = -.0004, SD= .53714), than in the CO (M = -.0239, SD = .51104) group, although the difference was not significant (MD = .02342, SEM = 0.4276, t_588_ = .548, p = .584, CI[-.06056, .10741]).


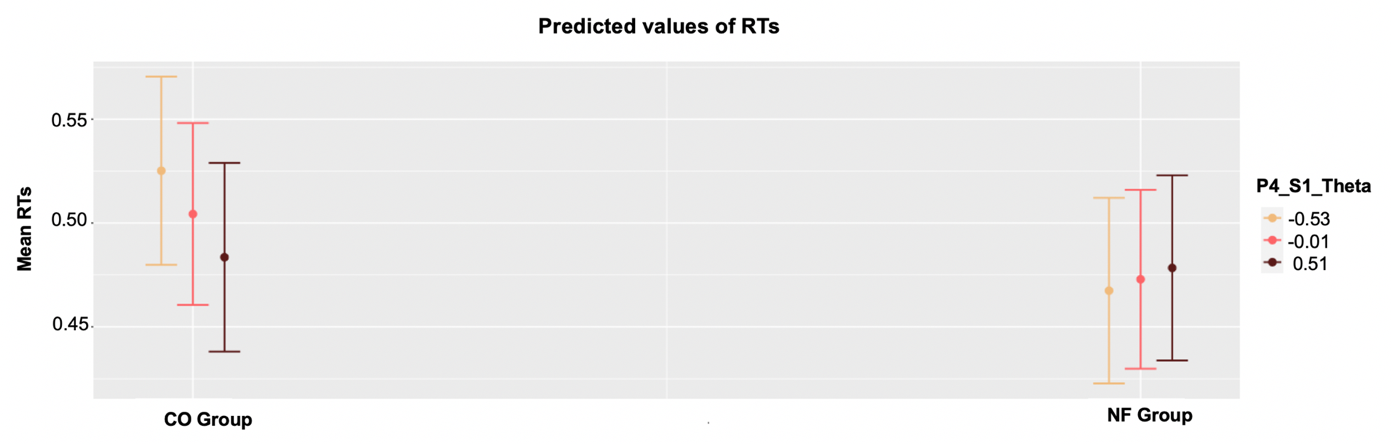


**Supplementary Fig. 5 Predicted values of RTs during encoding with interaction effects.** The figure shows results from the interaction H_1_ model based on restricted maximum likelihood (REML) identifying RTs as the target variable during encoding. Predicted values of RTs are represented for the NF group (right) and for the CO group (left) during encoding and in relation to the relative power of the right parietal theta at time window S1 during encoding. Groups are represented on the x-axis in relation to the relative power of theta and mean RTs on the y-axis. Standard error of the mean is denoted by the error bars. The brown line indicates higher values of theta power. The pink line indicates values around zero of theta power. The yellow line indicates lower values of theta power. Results showed that the interaction between group and right parietal theta at S1 was significant, indicating that an increase in theta power in the NF group was related to faster RTs (𝛽 = 0.05065, p = 0.001, CI[0.01870, 0.08260]).

**Supplementary Table 1.** **Model specification based on a post-hoc analysis and fixed effect coefficients (main effects and interactions) with related statistics**. Results from the interaction models H_1_ based on the restricted maximum likelihood (REML) are reported, respectively for accuracy and RTs during the encoding period. Fixed effect coefficients (main effects and interactions) and related estimates, p-values, lower and upper 95% confidence intervals (CI) are reported.

| Model specification: Linear mixed-effect model for accuracy during encoding | | | | |
| --- | --- | --- | --- | --- |
| Fixed effect coefficients | Estimate (coefficients) | p value | Lower (95% CI) | Upper (95% CI) |
| ‘Intercept’ | 55.649 | 0 | 51.168 | 60.129 |
| ‘Group_NF’ ^a^ | -0.43387 | 0.82518 | -4.29 | 3.4223 |
| ‘Reward’ ^b^ | 3.5267 | 0.0054715 | 1.0425 | 6.0108 |
| ‘Day’ ^c^ | 3.5209 | 5.2238e-12 | 2.5394 | 4.5023 |
| ‘P4_S2_Beta’ ^d^ | 8.8588 | 0.028525 | 0.93373 | 16.784 |
| ‘Group_NF : P4_S2_Beta’ ^e^ | 1.896 | 0.51258 | -3.7872 | 7.5793 |
| ‘Reward : P4_S2_Beta’ ^f^ | **-6.4747** | **0.023305 *** | **-12.066** | **-0.88352** |
| Day : P4_S2_Beta’ ^g^ | -1.02 | 0.29983 | -2.9505 | 0.91052 |
| Model specification: Linear mixed-effect model for RTs during encoding | | | | |
| Fixed effect coefficients | Estimate (coefficients) | p value | Lower (95% CI) | Upper (95% CI) |
| ‘Intercept’ | 0.6479 | 0 | 0.59003 | 0.70576 |
| ‘Group_NF’ | -0.03093 | 0.31429 | -0.09125 | 0.02939 |
| ‘Reward’ | -0.01222 | 0.1114 | -0.02729 | 0.00283 |
| ‘Day’ | -0.04594 | 2.82e-14 | -0.05750 | -0.03437 |
| ‘P4_S1_Theta’ ^h^ | -0.04024 | 0.04662 | -0.07987 | -0.00060 |
| ‘Group_NF : P4_S1_Theta’ ^i^ | **0.05065** | **0.00193 **** | **0.01870** | **0.08260** |
| Day : P4_S1_Theta’ ^j^ | 4.2859e-05 | 0.99414 | -0.01141 | 0.01149 |

^a^ Fixed effect coefficient “group” with 2 levels: NF group and CO group

^b^ Fixed effect coefficient “reward” with 2 levels: Reward and No-Reward conditions

^c^ Fixed effect coefficient “day” with 5 levels: day 1, day 2, day 3, day 4, and day 5

^d^ Fixed effect coefficient for beta frequency at channel P4, during the encoding period from 500 to 1000ms (S2)

^e^ Interaction between group and beta at P4 during S2

^f^ Interaction between reward conditions and beta at P4 during S2

^g^ Interaction between day and beta at P4 during S2

^h^ Fixed effect coefficient for theta frequency at channel P4, during the encoding period from 0 to 500ms (S1)

^i^ Interaction between group and theta at P4 during S1

^j^ Interaction between day and theta at P4 during S1

* Significant interaction between reward-anticipation and right parietal beta at S2 with P < 0.05

**Significant interaction between group and right parietal theta at S1 with P < 0.01

**Supplementary Table 2.** **Model specification based on a post-hoc analysis and fixed effect coefficients (main effects and interactions) with related statistics**. Results from the interaction models H_1_ based on the restricted maximum likelihood (REML) are reported, respectively for accuracy and RTs during the maintenance period (20s). Fixed effect coefficients and related coefficient estimates, p-values, lower and upper 95% confidence intervals (CI) are reported.

| Model specification: Linear mixed-effect model for RTs during maintenance | | | | |
| --- | --- | --- | --- | --- |
| Fixed effect coefficients | Estimate (coefficients) | p value | Lower (95% CI) | Upper (95% CI) |
| ‘Intercept’ | 0.64842 | 0 | 0.58926 | 0.70758 |
| ‘Group_NF’ | -0.03305 | 0.28875 | -0.09405 | 0.02804 |
| ‘Reward’ | -0.01002 | 0.1917 | -0.02509 | 0.00504 |
| ‘Day’ | -0.04520 | 4.2788e-13 | -0.05718 | -0.03323 |
| ‘P4_Beta’ ^a^ | -0.26277 | 0.00736 | -0.45468 | -0.07086 |
| ‘P3_Beta’ ^b^ | 0.22721 | 0.03270 | 0.018762 | 0.43565 |
| ‘Group_NF*P4_Beta’ ^c^ | 0.10527 | 0.14403 | -0.03606 | 0.24661 |
| ‘Group_NF*P3_Beta’ ^d^ | -0.09771 | 0.21942 | -0.25381 | 0.05839 |
| ‘Day*P4_Beta’ ^e^ | 0.03569 | 0.17937 | -0.01645 | 0.08784 |
| ‘Day*P3_Beta’ ^f^ | -0.02727 | 0.35987 | -0.08574 | 0.03118 |
| ‘P4_Beta*P3_Beta’ ^g^ | -0.05758 | 0.28995 | -0.16435 | 0.04919 |

^a^ Fixed effect coefficient for beta frequency at channel P4, during the entire maintenance period

^b^ Fixed effect coefficient for beta frequency at channel P3, during the entire maintenance period

^c^ Interaction between group and beta at P4, during the entire maintenance period

^d^ Interaction between group and beta at P3, during the entire maintenance period

^e^ Interaction between day and beta at P4, during the entire maintenance period

^f^ Interaction between day and beta at P3, during the entire maintenance period

^g^ Interaction between beta at P4 and beta at P3, during the entire maintenance period

**Supplementary Material 5 - Questionnaire post-NF-training**

Supplementary Material 5.1 – Appendix A

**Appendix A.** The twelve questions from the questionnaire post 5-days Neurofeedback training combined with the DMST are reported in German and English language.

| German Language | English Language |
| --- | --- |
| Wie gut fühlen Sie sich heute allgemein? | How do you feel today? |
| Wie anstrengend fanden Sie das Experiment? | How difficult was the experiment? |
| Wie fanden Sie Ihr Gedächtnis an Tag 1? | How do you estimate your memory on day 1? |
| Wie fanden Sie Ihr Gedächtnis an Tag 5? | How do you estimate your memory on day 5? |
| Fanden Sie es schwierig, die Aufgabenstellungen zu verstehen (z.B. das Neurofeedback zu kontrollieren oder sich an die Bilder zu erinnern)? | Did you find it difficult to understand the experimental instructions (e.g., to control the neurofeedback or to remember the images)? |
| Wie wichtig war es Ihnen, eine gute Leistung bei den Aufgaben zu zeigen? | How important was it for you, to show and to reach a good performance? |
| Fanden Sie es schwierig, sich an die Bilder zu erinnern? | Did you find it difficult to remember the images? |
| Haben Sie eine bestimmte Strategie für das Neurofeedback verwendet? | Did you use a specific strategy to perform the neurofeedback? |
| Wenn ja, welche? | If yes, which one? |
| Sind Ihnen Unterschiede von Tag 1 bis Tag 5 aufgefallen (z.B. Stimmung, Gedächtnis, Konzentration…)? | Did you find differences between day 1 and day 5 (e.g., concentration, memory, mood…)? |
| Haben Sie Verbesserungsvorschläge für dieses Experiment? | Do you have some suggestions to improve this experiment? |
| Wenn ja, welche? | If yes, which one? |

**Supplementary Material 6 - Study limitation**

In light of the method implemented and the obtained results, a few study limitations should be considered to potentially improve the outcome of future studies.

The neurofeedback (NF) training was performed based on the individual alpha range^13,14,15^, which was computed before the start of each training block. Therefore, the changes in the range of trained frequencies across blocks and training days might have affected participants’ ability to develop an effective strategy to control the NF signal. Future studies could try to implement a fixed NF frequency range across the training to evaluate whether this approach might improve the efficacy of the NF training.

The neurofeedback signal was generated based on a trial-by-trial normalization. Although this approach was applied to consider the potential modulation of alpha at baseline due to the NF training, the adjustment of the neurofeedback according to the trial-by-trial baseline might have increased the difficulty of the training and partially obscured the effect of the training. Indeed, a trial-by-trial normalization might be a rather less stable approach to baseline normalize the neurofeedback signal. Therefore, a fixed baseline could be implemented. This baseline can be recorded at the beginning of the entire training (e.g., in a familiarization session), or before the start of each training block (preferably in a session that is distant from the NF training, therefore not influenced by it).

In the offline analysis, a fixed frequency range was applied to alpha because also a fixed range was applied to the neighbouring frequencies theta and beta. The frequency range was 7-13 Hz. We were interested to investigate the training-related changes of alpha power across groups and conditions, and so a fixed range was needed. This choice might have affected the final results. Indeed, the NF training was based on individual alpha ranges across blocks and training days. Even though the 7-13 Hz is a good range to investigate the entire alpha spectrum, future studies could also investigate the alpha trend for each participant. This aspect was not possible in our study due to the incapability of recovering EEG recordings during the alpha range individualization sessions.

Moreover, we analysed the effect of NF-training normalization to the corresponding averaged-trial baseline (estimated for each day across the training). Considering the results from Study I, where a potential NF effect was observed on alpha suppression (e.g., Figure 3A reported in the manuscript), it might be possible that the choice of this baseline normalization approach might have made it more difficult to observe a difference in power across days. Therefore, future studies might apply a single baseline to potentially obtain a greater NF training effect. The choice of this baseline can be the average pre-trial baseline preceding the entire training, and thus not influenced by the potential effect of training.

Lastly, to observe a clear, stable, and reliable NF training effect, more than 5 sessions are needed. Indeed, initial improvements can be observed within 5 to 10 sessions, although for certain clinical conditions the number of sessions can range up to 40^16^. Besides, also the session frequency (e.g., number of sessions each week) should also be considered^17^.

**Supplementary Material 7 - References**

1. Faul F. G*Power. Hhu.de. Accessed June 30, 2022. http://www.gpower.hhu.de/
2. Biswas A, Ray S. Alpha neurofeedback has a positive effect for participants who are unable to sustain their alpha activity. eNeuro. 2019;6(4):ENEURO.0498-18.2019. doi:10.1523/ENEURO.0498-18.2019
3. Engelbregt HJ, Keeser D, van Eijk L, et al. Short and long-term effects of sham-controlled prefrontal EEG-neurofeedback training in healthy subjects. Clin Neurophysiol. 2016;127(4):1931-1937. doi:10.1016/j.clinph.2016.01.004
4. Farnia S, Abedi-Darzi S, Fattahi S, et al. The effect of beta and alpha neurofeedback on memory: A randomized, double-blind, sham-controlled, clinical trial. Iran J Psychiatry Behav Sci. 2017;11(2). doi:10.5812/ijpbs.7431
5. Walker EA, Redfern A, Oleson JJ. Linear mixed-model analysis to examine longitudinal trajectories in vocabulary depth and breadth in children who are hard of hearing. J Speech Lang Hear Res. 2019;62(3):525-542. doi:10.1044/2018_JSLHR-L-ASTM-18-0250
6. Rund BR, Barder HE, Evensen J, et al. Neurocognition and duration of psychosis: A 10-year follow-up of first-episode patients. Schizophr Bull. Published online 2015:sbv083. doi:10.1093/schbul/sbv083
7. Yuki O, Yul-Wan K, Seiji S. A linear mixed-effect model analysis of the effect of schizotypal personality traits on confidence in reality monitoring. Tohuku Psychologica Folia. 2019;77:83-90.
8. Magezi DA. Linear mixed-effects models for within-participant psychology experiments: an introductory tutorial and free, graphical user interface (LMMgui). Front Psychol. 2015;6:2. doi:10.3389/fpsyg.2015.00002
9. MATLAB 2018b (The MathWorks, Inc., Natick, Massachusetts, United States)
10. R Core Team. R: A Language and Environment for Statistical ## Computing. R Foundation for Statistical Computing; 2017. https://www.R-project.org/
11. Aiken, L. S., & West, S. G. (1991). Multiple regression: Testing and interpreting interactions. Sage Publications, Inc.
12. IBM Corp. Released 2018. IBM SPSS Statistics for Macintosh, Version 26.0
13. Babiloni C, Miniussi C, Babiloni F, et al. Sub-second“temporal atten-tion” modulates alpha rhythms. A high-resolution EEG study. BrainRes Cogn Brain Res. 2004;19:259-268.
14. Babiloni C, Stella G, Buffo P, et al. Cortical sources of resting state EEG rhythms are abnormal in dyslexic children. Clin Neurophysiol. 2012;123(12):2384-2391. doi:10.1016/j.clinph.2012.05.002
15. Klimesch W. EEG alpha and theta oscillations reflect cognitive and memory performance: a review and analysis. Brain Res Brain Res Rev. 1999;29(2-3):169-195. doi:10.1016/s0165-0173(98)00056-3.
16. Hammond DC. What is neurofeedback: An update. J Neurother. 2011;15(4):305-336. doi:10.1080/10874208.2011.623090
17. Domingos C, Peralta M, Prazeres P, Nan W, Rosa A, Pereira JG. Session frequency matters in neurofeedback training of athletes. Appl Psychophysiol Biofeedback. 2021;46(2):195-204. doi:10.1007/s10484-021-09505-3
